# Prevalence of dyslipidemia, hypertension and diabetes among tribal and rural population in a south Indian forested region

**DOI:** 10.1371/journal.pgph.0002807

**Published:** 2024-05-20

**Authors:** Sumanth Mallikarjuna Majgi, Yogish Channa Basappa, Srikanta Belagihalli Manjegowda, Savitha Nageshappa, Harshini Suresh, Giridhar R. Babu, Prashanth Nuggehalli Srinivas

**Affiliations:** 1 Department of Community Medicine, Mysore Medical College and Research Institute, Mysore, India; 2 Health Equity Cluster, Institute Public Health, Bengaluru, India; 3 Research Scientist II, Multi-Disciplinary Research Unit, Mysore Medical College and Research, Mysore, India; 4 Research Scientist-I, Multi-Disciplinary Research Unit, Mysore Medical College and Research, Mysore, India; 5 Research Fellow, SingHealth Duke-NUS Global Health Institute, Singapore, Singapore; 6 Department of Population Medicine, College of Medicine, QU Health, Qatar University, Doha, Qatar; Sciensano, BELGIUM

## Abstract

While NCDs are in rise globally, tribal and rural populations residing near to reserve forests with limited exposure to modern lifestyles may bear a unique burden. This study investigates the prevalence and risk factors of hypertension, diabetes, and dyslipidemia among these communities. We conducted a cross-sectional study between 2018 and 2020 in the forest-dwelling population of Chamarajanagar, India. Using multistage stratified sampling based on caste and remoteness, we enrolled 608 participants aged over 18 years, including 259 non-tribal and 349 tribal individuals. Data collection includes the administration of STEPS questionnaire and measurement of fasting blood sugar, lipid levels, and blood pressure. The prevalence of diabetes, hypertension, and dyslipidemia were 4.6%, 28.8%, and 85.7%, respectively, among the study population. We also found abnormal levels of low-density lipoprotein (LDL), high-density lipoprotein (HDL), Triglycerides (TGA), Total cholesterol (TC), and very low-density lipoprotein (VLDL)in 4.9%, 82.4%, 22.7%, 5.8%, and 7.4% of participants, respectively. Significant differences were observed in diabetes, LDL abnormality, TGA abnormality, VLDL abnormality, and TC abnormality, but not in hypertension, dyslipidemia, or HDL abnormality, across the Socio Geographic Discrimination Index. We found a significant difference in diabetes and HDL abnormality, but not in hypertension, dyslipidemia, LDL abnormality, TGA abnormality, TC abnormality, or VLDL abnormality, between tribal and non-tribal populations living in the forest-dwelling area. Waist circumference was a significant independent predictor of diabetes among tribal participants, while wealth index, age, and waist circumference were significant predictors of hypertension. There were no significant predictors for dyslipidemia among tribal participants. Our study suggests that tribal population living in a remote area are at a lower risk of developing diabetes compared to non-tribal populations living in the same geographic area. However, the prevalence of hypertension and dyslipidemia among tribal populations remains high and comparable to that of the general population.

## Introduction

Globally, non-communicable diseases (NCDs) account for 41 million death each year, equivalent to 74% of all-deaths worldwide, and 17 million premature deaths before the age of 70 [1]. Globally, there are 537 million adults (20–70 years) are livining with diabetes in 2021, which is likely to increase to 783 million by 2045 [2]. Similarly prevalence of hypertension globally was 24% in 2019, which is likely to increase to 29% by 2025 [3]. In India, over the years (1990 to 2019), mortality (35.8% to 64.9%) and Disability-Adjusted Life Year (DALYs) (29.1% to 57.9%) loss have substantially increased from NCDs [4]. The increasing trends of deaths from cardiovascular disease are due to an increase in hypertension, diabetes, dyslipidemia, dietary and behavioural changes [5].

Once regarded as lifestyle diseases with higher prevalence in affluent households, NCDs are increasingly reported from rural communities [6]. However, due to poor data quality from routine health services and limited research among hard-to-reach populations in forested areas with tribal and remote rural communities, their prevalence and risk factors have not been well characterized in public health literature. This limits the scope for behavioural change and engaging appropriate health systems response for these populations.

Due to proximity with forested and remote areas, tribal and remote rural populations in these areas are often hard to reach. tribal communities constitute 8.6% (i.e.,104 million) of India’s population [7]. Proximity to nature, unique occupational profile, limited urbanization, and high endogamy could modify individual and community-level risk profiles of these populations. Some of these characteristics are changing in states like Karnataka in southern India. The last few decades have seen improvements in literacy and the demographic structure of tribal populations, bringing them closer to rural India [8]. Dietary changes including lesser dependence on subsistence millets and restrictions on meat protein from hunting due to changing conservation regimes and higher dependence on pre-dominantly cereals from food subsidies too could be playing a role in addition to pressures from urbanization and labour mobility to urban areas among younger tribal households and its influence on food and behaviour [9,10].

There are scarce efforts at characterizing the risk factors and epidemiology of NCDs, particularly among remote rural and tribal populations, due to geographical challenges in organizing surveys and limited research infrastructure in forested and remote areas. Although several studies have examined the prevalence of hypertension (HTN) and Diabetes among tribal communities, data on the prevalence of these conditions among tribal and non-tribal communities residing in and around the forested area remains unavailable [6,11–15]. Although several meta-analyses have been conducted on diabetes and hypertension among tribal populations, none have been conducted using a systematic sampling effort among tribal populations in southern Karnataka [15–18]. In this study, researchers working on improving tribal health collaborated with a national initiative to establish Multi-Disciplinary Research Units in Government Medical colleges to improve evidence-based action on tribal health concerning NCDs [19,20]. In this study, we estimated the prevalence of hypertension, dyslipidemia, and diabetes in a South Indian tribal region. We have measured the risk factors for these non-communicable diseases (NCDs) among the tribal and non- tribal population, including physical activity, dietary patterns, obesity, alcohol consumption, and tobacco use. Additionally, we have investigated potential differences in NCD prevalence based on geographic factors (disadvantage/advantage) within the tribal area and between tribal and non-tribal populations residing within the region.

## Materials and methods

### Study design and setting

**Fig 1:**
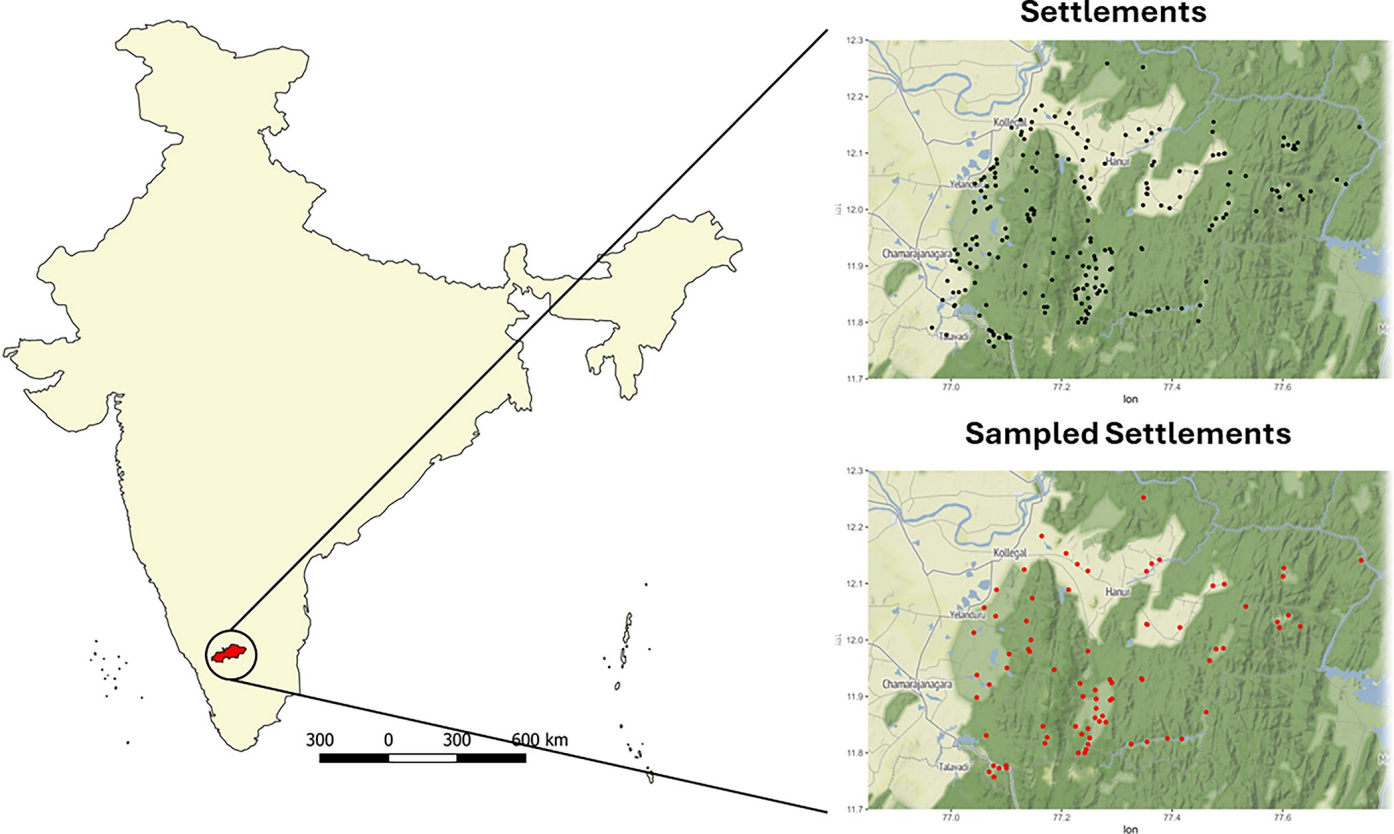
Location and hamlets/villages of Biligiriranga Hills (BR Hills) and MM hills in Karnataka. India base geography layer was retrieved from geoBoundaries (https://www.geoboundaries.org/). Settlements and sampled villages were plotted using R software using ggplot, with OpenStreetMap as the base layer.

A cross sectional study was conducted in the Chamarajanagar district of southern Karnataka, India. The district ranks among the least development in the state, occupying 22^nd^ position out of 30 districts on the human development index. Nearly half of the district is covered by forests, legally protected under India’s Wildlife Protection Act 1972, consisting of two tiger reserves Biligiriranga Hills (B R Hills) and Bandipur tiger reserves and Maleya Mahadeshwara (M M Hills) wildlife sanctuary [21]. According to the 2011 census around 11.8% of the district population, belong to Scheduled tribal (STs). Communities legally recognized as STs are entitled to affirmative action benefits in education and employment. A significant number of tribal communities residing in the BR hills and MM hills area (Fig 1) belong to the Soliga/Solega. These communities self-identify as Adivasi, which translates to "first citizens" in multiple Indian languages. The most recent census estimates the Soliga population to be around 36,000. The Soliga tribal might be one of the earliest inhabitants of this region and, along with several other tribal communities in southern Indiagenetic to Australian aboriginal populations [22]. We included human settlements located within BR Hills and MM Hills Forest areas, as well as settlements within a 5 km radius of the forest boundary. This combined area, encompassing both forest and adjacent villages, was called as the “tribal area" as which was mapped using QGIS Software [23]. Since Karnataka is not one among the states that statutorily notify specific areas astribal areas, this approach was chosen to facilitate sampling and ensure all relevant communities were represented in the study [24]. Several social and biomedical drivers of health inequities of tribal populations have been reported despite the protection of their rights under Article 342 of the Indian Constitution [25,26].

Sample size: We estimated a sample size of 384 for each sites, aiming for a total of 800 participants. Assuming an average prevalence of 20% for hypertension, diabetes and dyslipidemia (ranging from 3% to50%) and a relative precision of 20% [27–31], We used the standard sample size formula for cross-sectional/cohort study design on OpenEpi [32].

Due to the Covid-19 pandemic, we stopped data collection at 568 participants for both sites, prioritizing the safety of field staff and research participants over further data collection.

### Sampling strategy

Operational definition of tribal area in our study includes villages/settlements within BR Hills and MM Hills forests and buffer zone of 5 km distance from the edge of the forest boundary defined by forest department.

**Fig 2:**
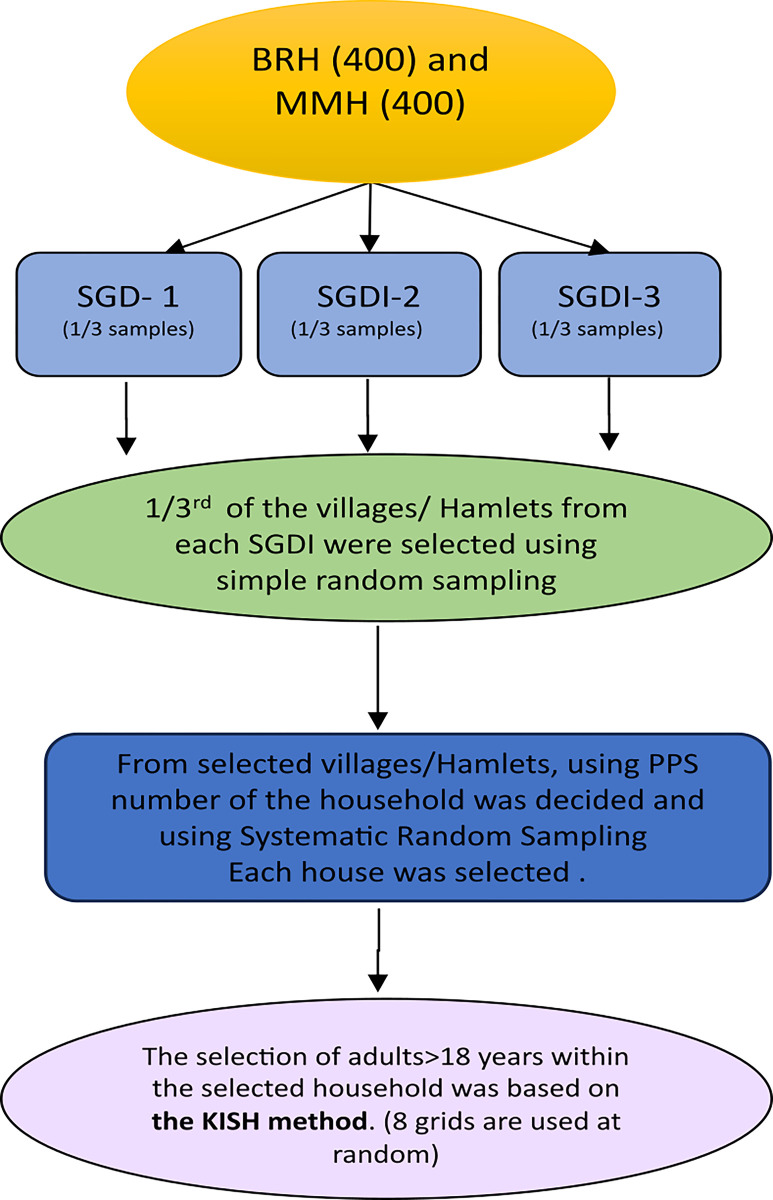
Sampling methodology.

This study is a nested study within the “Towards Health Equity and Transformative Action on tribal health (THETA)” project, a multi-site research study on tribal health inequities[19]. A multistage stratified sampling methodology (Fig 2) was used, with villages at both MM Hills and BR Hills selected at the first sampling stage from 2011 Census Enumeration Areas and mapped using QGIS software.

We identified clusters of villages/settlements that shared socio-geographical attributes using principal component analysis, with intra-cluster homogeneity and inter-cluster heterogeneity for geography. These clusters were the primary strata, and three such clusters were identified per site covering groups of villages in remote (core forest area), somewhat remote villages at the edge/outside the forest, villages that may be inside the forest but well-connected by all-weather roads, and groups of relatively well-connected villages in the plain (non-remote) [19].

For each group, one-third of the sample size was allocated, with one-third of the villages/hamlets randomly selected as a secondary sampling unit within each group. The number of tribal and non-tribal households within each of these selected villages/hamlets was selected based on the Probability Proportion to Size (PPS) population. Every Nth household was selected based on the number of households, and if the approached household members were unavailable or did not consent to participate, the next household was chosen. Only one individual was interviewed randomly within each selected household using the Kish table method [33].

To ensure that each substratum had an equal representation, the total sample size required for this study was 840 participants. This translated into 70 participants in each subgroup of the tribal and non-tribal groups of each remoteness category (non-remote, somewhat remote, and remote). There are approximately 135 villages/settlements in BR Hills and 79 villages/settlements in MM Hills. Using the sampling criteria described above, we selected 49 villages/settlements (7 remote, 12 somewhat remote, and 30 non-remote) in the BR Hills area and 38 villages/settlements (11 remote, 12 somewhat remote, and 15 non-remote) in the MM Hills area.

We enumerated family members of the selected households based on their age and selected a respective number from the KISH grid to identify the participant within the household. However, this method was not always successful, as adult male participants either refused to participate or were not available despite 2–3 visits. In such cases, we sought a replacement from the household, typically an adult female. We excluded household members who were severely ill/bed-ridden, could not participate in the study, and pregnant or lactating women.

### Data collection

Local data enumerators, who were trained for the study. We invited adult (above 18 years) potential participants to participant in the study from the household survey. Informed verbal consent was obtained from each participant and recorded in data collection app. The detailed ethics procedures are outlined in ethics section. We collected information on socio-demographic variables, such as age, gender, occupation, income, marital status, wealth index, and other relevant factors, using tools published in the protocol. We also obtained information on non-communicable disease risk factors, including physical activity, anthropometry, diet, and family history of diabetes and hypertension, based on the Integrated Disease Surveillance Project (IDSP) NCD risk factor survey [19,34,35]. We measured the food frequency and quantity of vegetables, fruits, and other commonly eaten foods on the days they were consumed. Physical activity was measured using Integrated disease surveillance project (IDSP) questionnaire, which is the modified version of international physical activity questionnaire. Based on the type of the type of the type and frequency of the activity, Metabolic equivalents (MET) scores were calculated and classified further based on the total score as low activity, moderate activity and vigorous activity based on international physical activity questionnaire [34,36,37]. Anthropometry measurements, including BMI (Body Mass Index), waist circumference, and waist-to-hip ratios, were also measured and calculated using standard procedures [38]. We measured weight using an Omron weighing machine with a sensitivity of 100 grams and height using a mobile stadiometer with a sensitivity of 1 centimeter; BMI was classified according to the Asian classification [39]. Waist Circumference and Waist Hip Ratio was measured as per standard procedure, Individuals with WC of ≥90 cm for men and ≥80 cm for women were considered cutoff points for defining abdominal obesity [36].

Blood pressure measurements were taken using an Omron BP apparatus (HEM 7120, 2017IN). A minimum of two readings were taken at intervals of at least 3 to 5 minutes, and the average of those readings represented the person’sblood pressure. If there was a >10 mm Hg difference between the first and second readings, additional readings were obtained, and the average of these multiple readings was used. Standardization of equipment was done before the initiation of the survey.

**Fig 3:**
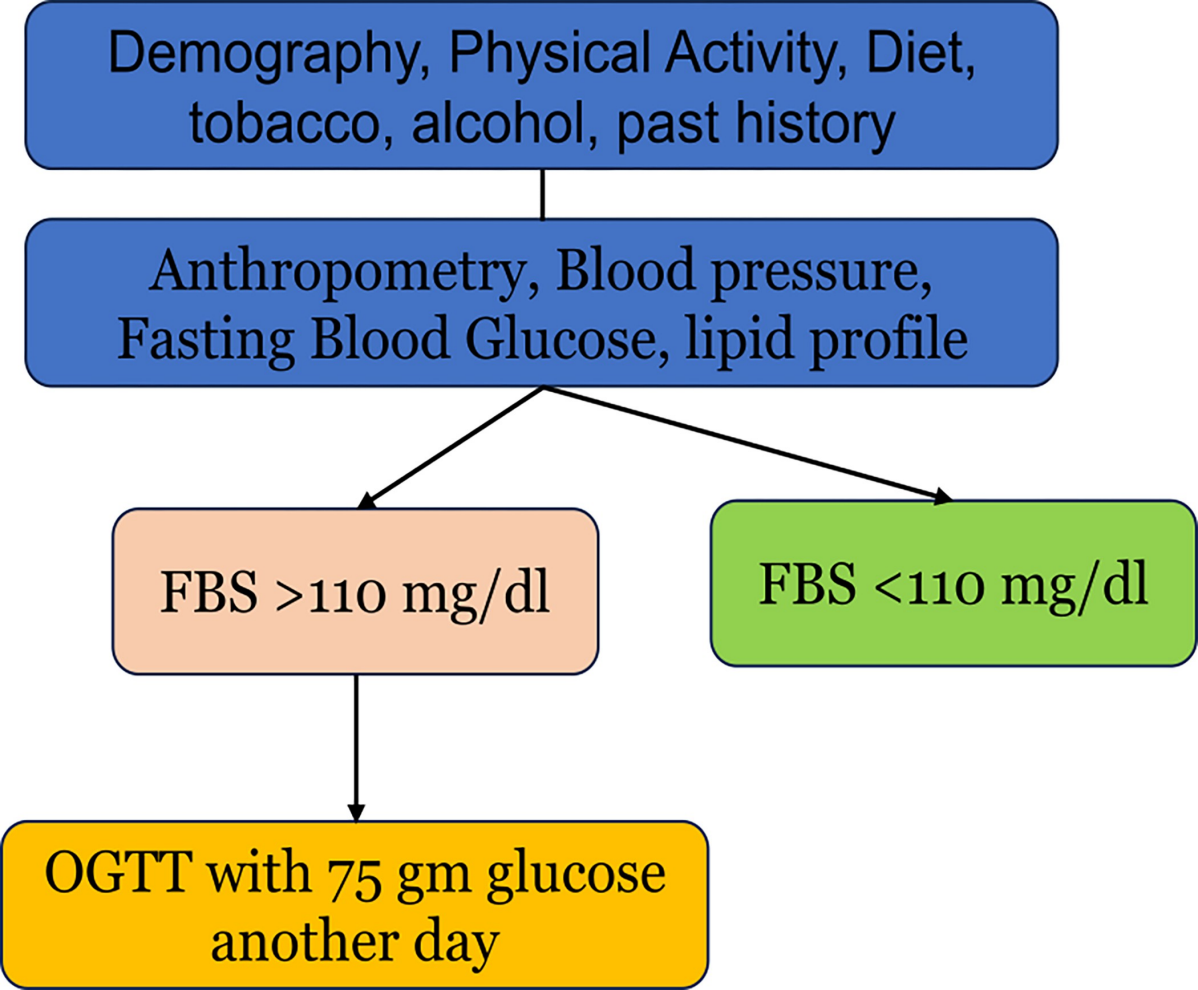
Sequence of activities leading to blood tests.

Around 5 ml of venous blood was collected from participants. After 30 min of sedimentation, it was centrifuged at the collection point (common point hear household) at 3000 RPM for 15 min and processed for whole blood and serum and stored under refrigeration till further use (stored at -20 c). Every fifteen days, the samples were transported to the testing centre (MMCRI, MRU) with a cold chain (-20 to -140C, using dry ice) without thawing and freezing. Fasting blood glucose (FBS) was measured using the Glucose oxidase method by autoanalyzer (Roche Diagnostics, Germany). OGTT was done only for those >110 FBS for logistic and feasibility reasons (Fig 3). Serum cholesterol, HDL, LDL, and triglycerides were measured on fasting samples using an enzyme-based method and expressed as mg/dl of serum [40]. The measurement was made using an auto-analyser using an enzymatic kit from Roche. Hypercholesterolemia was defined as serum cholesterol of ≥200 mg/dl. Classification of lipid profile was according to NCEP-ATP III classification [41]. Also, abnormality in any one of the components of lipid profile was considered as dyslipidemia. Data collection was conducted between 1^st^ November 2018 to 20^th^ March 2020.

### Definition

**Hypertension:** Raised Blood pressure/ Hypertension is (systolic blood pressure (SBP) ≥ 140 and/or diastolic blood pressure (DBP) ≥ 90 mmHg or currently on medication for raised Blood pressure/ Hypertension) [42,43]. Also, those with known history of hypertension with or without on treatment are also considered as hypertension.

**Prediabetes:** As per the recommendations of the World Health Organization, fasting FBS of <110 mg/dl, 110–125 mg/dl, and ≥126 mg/dl were considered as normal, impaired fasting glucose (prediabetes) and diabetic, respectively [40].

**Diabetes:** Those with known diagnosis of diabetes mellitus with or without on treatment; or those participants whose OGTT value are in diabetic range (FBS≥126 mg/dl and/or 2hr 75 g Glucose value ≥200mg/dl) [40].

**Metabolic Equivalent (MET)**: MET is the ratio of a person’s working metabolic rate relative to the resting metabolic rate. One MET is defined as the energy cost of sitting quietly, and is equivalent to a caloric consumption of 1 kcal/kg/hour (S1 Text) [36,37].

Dyslipidemia: Dyslipidemia is characterized by an elevation of serum total cholesterol (TC), reduced serum high density lipoprotein cholesterol (HDL) and low density lipoprotein cholesterol (LDL) or triglycerides (TG) concentration [36,44]

### Data analysis

Data were summarized into numbers, percentages, mean, and standard deviation. The Chi-square test was applied to assess the difference in proportions between two independent groups. Wherever necessary, the data were subjected to multivariable analysis using binary logistic regression. Those variables with 0.2 in univariate analysis were added in the multivariable model. If P<0.05, it is said to be statistically significant. SPSS (Statistical Package for the Social Sciences) version 21 software (IBM Corp Armonk, NY, USA) was used for statistical analysis.

### Ethics statement

Study information was provided using a recorded video in kannada local dialect, and a written participation information sheet which includes purpose of the study, risks and benefits. After explaining the information sheet, oral consent was obtained, recorded, and stored in the Fulcrum offline data collection app. Witnessing was provided by either family members or community leaders [45].

Based on piloting, we found this to be an effective means of obtaining consent. It was not perceived as threatening (unlike written consent on paper forms) and was more culturally acceptable. We informed our ethics committee about the potential risks associated with this approach, including the possible exclusion of various participants if mandatory written consent was imposed. Considering that we collaborated with local partners, community-based organizations, and gate keepers. We concluded that the implementation of a recorded verbal consent in the presence of a witness could be an effective way of obtaining informed consent. The study received ethics clearance from the institutional ethics committee of Mysore Medical College and Research Institute (dated 2^nd^ August 2018) and for THETA project which received ethics clearance from the institutional ethics committee of Institute of Public Health, Bangalore (Study ID IEC-FR/03/2018 vide IEC letter number IPH/18-19/E/226 dated 5th July 2018 valid till July 2019; renewed vide IEC IPH/19-20/E/183 valid till March 2020). Both ethics committees approved the oral consent process.

## Results

Due to the COVID-19 pandemic, the survey achieved a 76% coverage rate (608 participants out of 800 planned participants) due to the COVID-19 pandemic. Among these participants, the response rate was 93.4% (568 out of 608) (reasons are temporary migration, unwillingness to give a blood sample, and prioritizing someone else than themselves in the family). 17% of participants (98 individuals) were replaced in KISH grid. Among those with data, only 568 completed all sections (93% completion rate). The remaining participants missed either some of the survey questions (28) or the blood sample data (12). The sample distribution across remoteness levels was: 187 from non-remote, 191 from somewhat remote, and 230 from remote areas.

The final dataset has a slightly higher proportion of Tribal (59%) and remote participants (41%) in overall. An about 51.6% of sampled population groups falls in the age group of 26 to 40 year, followed by 20.6% from 41 to 55 years. Over two-thirds of the participants were female, most study participants were married (84.2%), Almost half (49.2%) of the participants were reported being literate overall. Majority were married and mostly do casual wage labour.

The age, gender, marital status, literacy distribution was no different among tribal and non tribal communities, however, there were more casual wage labourer in tribal, more self-employed participants in the non tribal participants and higher proportion of fourth and highest quintile of wealth index in non-tribal compared to tribal participants (Table 1).

**Table 1:** Demography characteristics of the study participants.

		Non Tribal (233)		Tribal (335)		Total (568)	
		n	%	n	%	n	%
Remoteness	Non remote	56	24.0%	120	35.8%	176	31.0%
	Somewhat remote	82	35.2%	94	28.1%	176	31.0%
	Remote	95	40.8%	121	36.1%	216	38.0%
Age category (in years)	<25	36	15.5%	76	22.7%	112	19.7%
	26–40	127	54.5%	173	51.6%	300	52.8%
	41–55	48	20.6%	66	19.7%	114	20.1%
	> = 56	22	9.4%	20	6.0%	42	7.4%
Gender	Male	75	32.2%	106	31.6%	181	31.9%
	Female	158	67.8%	229	68.4%	387	68.1%
Marital status	Married	204	87.6%	275	82.1%	479	84.3%
	Never married	12	5.2%	32	9.6%	44	7.7%
	Separated/Divorced	0	0.0%	3	0.9%	3	0.5%
	Widowed	17	7.3%	25	7.5%	42	7.4%
literacy	Illiterate	114	48.9%	179	53.4%	293	51.6%
	Literate	119	51.1%	156	46.6%	275	48.4%
Occupation	Casual wage labourer	95	40.8%	173	51.6%	268	47.2%
	Housewife	75	32.2%	92	27.5%	167	29.4%
	Not applicable/Not Employed/Student/others	9	3.9%	15	4.5%	24	4.2%
	Salaried employee	7	3.0%	18	5.4%	25	4.4%
	Self employed	47	20.2%	37	11.0%	84	14.8%
Wealth Index	Lowest	41	17.6%	78	23.3%	119	21.0%
	Second	24	10.3%	100	29.9%	124	21.8%
	Middle	45	19.3%	91	27.2%	136	23.9%
	Fourth	53	22.7%	43	12.8%	96	16.9%
	Highest	70	30.0%	23	6.9%	93	16.4%

We found no statistically significant differences in alcohol use, vegetable use, use of outside food, use of additional salt, use of sweet drinks, and physical activity levels between tribal and non-tribal participants (see Table 2). Inadequate use of vegetables and fruits was very high (>96%) in both Tribal and non-tribal participants, and the use of fried and processed foodswas high (~10%). However, there were statistically significant differences in waist circumference, BMI, total body fat, visceral fat, body muscle, aerated drink use (although the quantum of difference was not significantly higher), red meat consumption, use of chicken, smoking, and use of smokeless tobacco between tribal and non-tribal participants.

**Table 2:** Distribution of Behavioural risk factors of the study participants (with 95%CI).

		Non Tribal(233)		Tribal(335)		Total		
		n	%(95%CI)	n	%(95%CI)	n	%(95%CI)	p
Ever used smoked tobacco		57	24.5(19.3–30.3)	137	40.9(35.7–46.2)	194	34.2(30.3–38.1)	<0.001
Ever used smokeless tobacco		22	9.4(6.2–13.7)	62	18.5(14.6–22.9)	84	14.8(12.1–17.9)	0.003
Current tobacco use		48	20.6(15.8–26.1)	117	34.9(30–40.1)	165	29(25.4–32.9)	<0.001
Daily tobacco use		47	20.2(15.4–25.7)	112	33.4(28.5–38.6)	159	28(24.4–31.8)	<0.001
ever used alcohol.		36	15.5(11.2–20.5)	59	17.6(13.8–22)	95	16.7(13.8–20)	0.5
HRD *	>6SD	9	3.9(1.9–6.9)	19	5.7(3.6–8.5)	28	4.9(3.4–6.9)	0.6
Physical activity level	Low	8	3.4(1.6–6.4)	9	2.7(1.3–4.8)	17	3(1.8–4.6)	0.9
	Moderate	31	13.3(9.4–18.1)	43	12.8(9.6–16.7)	74	13(10.4–16)	
	High	194	83.3(78.1–87.6)	283	84.5(80.3–88.1)	477	84(80.8–86.8)	
Inadequate Fruits and Vegetables		225	96.6(93.6–98.4)	328	97.9(95.9–99.1)	553	97.4(95.8–98.4)	0.3
Added salt use frequency	Never	181	77.7(72–82.7)	252	75.2(70.4–79.6)	433	76.2(72.6–79.6)	0.9
	Rarely	20	8.6(5.5–12.7)	30	9(6.2–12.4)	50	8.8(6.7–11.3)	
	Sometime	12	5.2(2.8–8.6)	23	6.9(4.5–9.9)	35	6.2(4.4–8.4)	
	often	16	6.9(4.1–10.7)	22	6.6(4.3–9.6)	38	6.7(4.9–9)	
	Always	4	1.7(0.6–4)	8	2.4(1.1–4.5)	12	2.1(1.2–3.6)	
Processed food use frequency	Never	44	18.9(14.3–24.3)	58	17.3(13.6–21.6)	102	18(15–21.3)	0.4
	Rarely	41	17.6(13.1–22.9)	44	13.1(9.8–17.1)	85	15(12.2–18.1)	
	Sometime	71	30.5(24.8–36.6)	120	35.8(30.8–41.1)	191	33.6(29.8–37.6)	
	often	53	22.7(17.7–28.4)	86	25.7(21.2–30.5)	139	24.5(21.1–28.1)	
	Always	24	10.3(6.9–14.7)	27	8.1(5.5–11.3)	51	9(6.8–11.5)	
Outside meals per week	Nil	154	66.1(59.8–71.9)	217	64.8(59.6–69.7)	371	65.3(61.3–69.1)	0.9
	1–3 per week	72	30.9(25.2–37)	107	31.9(27.1–37.1)	179	31.5(27.8–35.4)	
	>3 per week	7	3(1.4–5.8)	11	3.3(1.8–5.6)	18	3.2(2–4.9)	
Consumption of fried food	Never	21	9(5.8–13.2)	16	4.8(2.9–7.5)	37	6.5(4.7–8.8)	0.2
	Rarely	27	11.6(8–16.2)	42	12.5(9.3–16.4)	69	12.1(9.7–15)	
	Sometime	69	29.6(24–35.7)	85	25.4(20.9–30.2)	154	27.1(23.6–30.9)	
	often	94	40.3(34.2–46.7)	151	45.1(39.8–50.4)	245	43.1(39.1–47.2)	
	Always	22	9.4(6.2–13.7)	41	12.2(9.1–16.1)	63	11.1(8.7–13.9)	
Consumption of red meat	Never	111	47.6(41.3–54)	38	11.3(8.3–15.1)	149	26.2(22.7–30)	<0.001
	Rarely	16	6.9(4.1–10.7)	30	9(6.2–12.4)	46	8.1(6.1–10.6)	
	Sometime	22	9.4(6.2–13.7)	64	19.1(15.2–23.6)	86	15.1(12.4–18.3)	
	often	74	31.8(26–37.9)	181	54(48.7–59.3)	255	44.9(40.8–49)	
	Always	10	4.3(2.2–7.5)	22	6.6(4.3–9.6)	32	5.6(4–7.8)	
Consumption of chicken	Never	109	46.8(40.4–53.2)	37	11(8–14.7)	146	25.7(22.2–29.4)	<0.001
	Rarely	4	1.7(0.6–4)	11	3.3(1.8–5.6)	15	2.6(1.6–4.2)	
	Sometime	22	9.4(6.2–13.7)	54	16.1(12.5–20.3)	76	13.4(10.8–16.4)	
	often	87	37.3(31.3–43.7)	207	61.8(56.5–66.9)	294	51.8(47.7–55.9)	
	Always	11	4.7(2.5–8)	26	7.8(5.3–11)	37	6.5(4.7–8.8)	
Consumption of aerated drinks	Never	66	28.3(22.8–34.4)	125	37.3(32.3–42.6)	191	33.6(29.8–37.6)	0.03
	Rarely	54	23.2(18.1–28.9)	75	22.4(18.2–27.1)	129	22.7(19.4–26.3)	
	Sometime	42	18(13.5–23.3)	69	20.6(16.5–25.2)	111	19.5(16.4–23)	
	often	60	25.8(20.5–31.6)	58	17.3(13.6–21.6)	118	20.8(17.6–24.3)	
	Always	11	4.7(2.5–8)	8	2.4(1.1–4.5)	19	3.3(2.1–5.1)	
Consumption of sweet drink	Never	58	24.9(19.7–30.7)	90	26.9(22.3–31.8)	148	26.1(22.6–29.8)	0.9
	Rarely	53	22.7(17.7–28.4)	74	22.1(17.9–26.8)	127	22.4(19.1–25.9)	
	Sometime	47	20.2(15.4–25.7)	64	19.1(15.2–23.6)	111	19.5(16.4–23)	
	often	65	27.9(22.4–33.9)	89	26.6(22.1–31.5)	154	27.1(23.6–30.9)	
	Always	10	4.3(2.2–7.5)	18	5.4(3.3–8.2)	28	4.9(3.4–6.9)	
BMI category	Underweight(<18.5)	79	34.1(28.2–40.3)	166	49.7(44.4–55)	245	43.3(39.2–47.4)	<0.001
	Normal (18.5–23)	80	34.5(28.6–40.8)	126	37.7(32.7–43)	206	36.4(32.5–40.4)	
	Overweight (23.1–25)	25	10.8(7.3–15.2)	23	6.9(4.5–10)	48	8.5(6.4–11)	
	Obese (>25)	48	20.7(15.9–26.2)	19	5.7(3.6–8.6)	67	11.8(9.4–14.7)	
Waist Circumference	Increased	94	41(34.8–47.5)	62	18.6(14.7–23.1)	156	27.8(24.2–31.6)	<0.001
Visceral Fat	High	30	13(9.2–18)	16	4.9(2.97–7.83)	46	8.3(6.21–10.8)	<0.001

*HRD-High Risk Drinking, >6 standard drink (SD) per sitting.

Among tribal participants, there was a higher prevalence of smoking (ever-smoked tobacco), daily tobacco use, smokeless tobacco use, lower obesity, and higher under-nutrition. Physical activity levels were similar in both tribal and non-tribal participants. Of the 109participants who currently smoke, 98 (88.9%) smoke beedis (local cigarettes) only, while 6(5.5%) smoke both beedis and cigarettes, and 5 (4.6%) smoke only cigarettes. Of the 66who use smokeless tobacco, 58(87.9%) use plain tobacco for chewing, while 6 (9.1%) chew tobacco with pan (a mixture of beetle leaves, arecanut, and lime with other masala), and 1 (1.5%) each use snuff, plain tobacco, and tobacco with pan.

Nearly 48% of male participants have not ever used alcohol, 36% reported being currently used, and 16% reported being past users. Nearly 16% of male participants reported high-risk drinking. The mean age at initiation of alcohol use was 23±7 years (range 10–42). Approximately 28% of participants had high waist circumference, 8.3% had high visceral fat, and 56.8% had high waist-to-hip ratio. Although nearly 20% of participants were overweight or obese by BMI, nearly half (43%) had under-nutrition. There were significant high obesity in terms of both BMI and waist circumference among non-tribal participants compared to tribal participants.

The abdominal obesity was significantly higher among female 32.7% (95% CI 28.2–37.5) compared to male 17.2% (95% CI 12.3–23.2), and the similar difference was seen among non-Tribal population also male 5.7% (95% CI 2.4–11.3) Female 24.7% (95% CI 19.4–30.6), however no such significant difference was seen among Tribal population male 33.8% (95% CI 23.8–45), female 44.5% (95% CI 36.9–52.4).

There was a significant higher proportion of ever smoked and daily tobacco use in remote area compared to non-remote and somewhat remote areas. However, inadequate use of fruits and vegetables was high irrespective of remoteness. The frequency of chicken use and red meat consumption was significantly higher in remote locations. Obesity was significantly lower in remote participants (3.7% vs 14.9% and 18.8%), with significant higher underweight (51.6% vs 41.1% and 35.2%) compared to somewhat remote and non-remote location. There were no significant differences in physical activity levels across the remoteness (see Table 3).

**Table 3:** Comparison of Behavioural risk factors of the study participants across remoteness.

		Non remote(176)		Somewhat remote(176)		Remote(216)		
		n	% (95%CI)	n	% (95%CI)	n	% (95%CI)	p
Ever used smoked tobacco		47	26.7(20.6–33.6)	59	33.5(26.9–40.7)	88	40.7(34.3–47.4)	0.014
Ever used smokeless tobacco		23	13.1(8.7–18.6)	21	11.9(7.8–17.3)	40	18.5(13.8–24.1)	0.14
Daily tobacco use		39	22.2(16.5–28.7)	47	26.7(20.6–33.6)	73	33.8(27.7–40.3)	0.035
ever used alcohol		28	15.9(11.1–21.8)	24	13.6(9.2–19.3)	43	19.9(15–25.6)	0.24
HRD *	>6SD	10	5.7(3–9.8)	9	5.1(2.6–9.1)	9	4.2(2.1–7.5)	0.7
Physical activity level	Low	9	5.1(2.6–9.1)	6	3.4(1.4–6.9)	2	0.9(0.2–2.9)	0.1
	Moderate	27	15.3(10.6–21.2)	23	13.1(8.7–18.6)	24	11.1(7.4–15.8)	
	High	140	79.5(73.1–85)	147	83.5(77.5–88.4)	190	88.0(83.1–91.8)	
Inadequate fruits and vegetables	Yes	172	97.7(94.7–99.2)	168	95.5(91.6–97.8)	213	98.6(96.3–99.6)	0.15
Added salt use frequency	Never	137	77.8(71.3–83.5)	132	75(68.2–81)	164	75.9(69.9–81.3)	0.33
	Rarely	14	8(4.6–12.6)	12	6.8(3.8–11.3)	24	11.1(7.4–15.8)	
	Sometime	11	6.3(3.4–10.6)	13	7.4(4.2–12)	11	5.1(2.7–8.6)	
	Often	8	4.5(2.2–8.4)	17	9.7(6–14.7)	13	6(3.4–9.8)	
	Always	6	3.4(1.4–6.9)	2	1.1(0.2–3.6)	4	1.9(0.6–4.3)	
Processed food use frequency	Never	42	23.9(18–30.6)	31	17.6(12.5–23.7)	29	13.4(9.4–18.4)	0.2
	Rarely	26	14.8(10.1–20.6)	24	13.6(9.2–19.3)	35	16.2(11.8–21.6)	
	Sometime	58	33(26.3–40.1)	57	32.4(25.8–39.5)	76	35.2(29–41.7)	
	Often	35	19.9(14.5–26.2)	51	29(22.7–36)	53	24.5(19.2–30.6)	
	Always	15	8.5(5.1–13.3)	13	7.4(4.2–12)	23	10.6(7.1–15.3)	
outside meals per week	Nil	115	65.3(58.1–72.1)	112	63.6(56.4–70.5)	144	66.7(60.2–72.7)	0.3
	1–3 per week	52	29.5(23.2–36.6)	58	33(26.3–40.1)	69	31.9(26–38.4)	
	>3 per week	9	5.1(2.6–9.1)	6	3.4(1.4–6.9)	3	1.4(0.4–3.7)	
Consumption of fried food	Never	13	7.4(4.2–12)	8	4.5(2.2–8.4)	16	7.4(4.5–11.5)	0.1
	Rarely	16	9.1(5.5–14)	24	13.6(9.2–19.3)	29	13.4(9.4–18.4)	
	Sometime	42	23.9(18–30.6)	40	22.7(17–29.3)	72	33.3(27.3–39.8)	
	Often	83	47.2(39.9–54.5)	84	47.7(40.4–55.1)	78	36.1(29.9–42.7)	
	Always	22	12.5(8.2–18)	20	11.4(7.3–16.7)	21	9.7(6.3–14.2)	
Consumption of red meat	Never	31	17.6(12.5–23.7)	27	15.3(10.6–21.2)	91	42.1(35.7–48.8)	<0.001
	Rarely	12	6.8(3.8–11.3)	17	9.7(6–14.7)	17	7.9(4.8–12)	
	Sometime	31	17.6(12.5–23.7)	28	15.9(11.1–21.8)	27	12.5(8.6–17.4)	
	Often	87	49.4(42.1–56.8)	98	55.7(48.3–62.9)	70	32.4(26.4–38.9)	
	Always	15	8.5(5.1–13.3)	6	3.4(1.4–6.9)	11	5.1(2.7–8.6)	
Consumption of chicken	Never	29	16.5(11.6–22.5)	30	17(12–23.1)	87	40.3(33.9–46.9)	<0.001
	Rarely	5	2.8(1.1–6.1)	6	3.4(1.4–6.9)	4	1.9(0.6–4.3)	
	Sometime	27	15.3(10.6–21.2)	21	11.9(7.8–17.3)	28	13(9–17.9)	
	Often	97	55.1(47.7–62.3)	112	63.6(56.4–70.5)	85	39.4(33–46)	
	Always	18	10.2(6.4–15.3)	7	4(1.8–7.6)	12	5.6(3.1–9.2)	
Consumption of aerated drinks	Never	73	41.5(34.4–48.8)	59	33.5(26.9–40.7)	59	27.3(21.7–33.5)	0.001
	Rarely	52	29.5(23.2–36.6)	29	16.5(11.6–22.5)	48	22.2(17.1–28.1)	
	Sometime	23	13.1(8.7–18.6)	38	21.6(16–28.1)	50	23.1(17.9–29.1)	
	Often	25	14.2(9.6–19.9)	43	24.4(18.5–31.2)	50	23.1(17.9–29.1)	
	Always	3	1.7(0.5–4.5)	7	4(1.8–7.6)	9	4.2(2.1–7.5)	
Consumption of sweet drink	Never	57	32.4(25.8–39.5)	44	25(19–31.8)	47	21.8(16.7–27.6)	0.5
	Rarely	38	21.6(16–28.1)	36	20.5(15–26.9)	53	24.5(19.2–30.6)	
	Sometime	31	17.6(12.5–23.7)	38	21.6(16–28.1)	42	19.4(14.6–25.1)	
	Often	44	25(19–31.8)	48	27.3(21.1–34.2)	62	28.7(23–35)	
	Always	6	3.4(1.4–6.9)	10	5.7(3–9.8)	12	5.6(3.1–9.2)	
BMI category	Underweight(<18.5)	62	35.2(28.5–42.5)	72	41.1(34–48.5)	111	51.6(45–58.2)	<0.001
	Normal (18.5–23)	65	36.9(30.1–44.2)	65	37.1(30.2–44.5)	76	35.3(29.2–41.9)	
	Overweight (23.1–25)	16	9.1(5.5–14)	12	6.9(3.8–11.3)	20	9.3(6–13.7)	
	Obese (>25)	33	18.8(13.5–25)	26	14.9(10.2–20.7)	8	3.7(1.8–6.9)	
Waist Circumference	Increased	55	31.8(25.2–39)	59	33.9(27.2–41.2)	42	19.5(14.7–25.2)	0.003
Visceral Fat	10-14(high)	20	11.4(7.4–16.8)	21	12.1(7.9–17.5)	5	2.4(0.0.9–5.1)	<0.001

*HRD-High Risk Drinking, >6 standard drink (SD) per sitting.

There is a significant difference in high LDLs, total cholesterol and VLDL across the socio-geographic disadvantage index. Those in remote areas are less likely to develop these abnormalities (Table 4).

**Table 4:** Comparison of NCD of the study participants across remoteness.

	Non remote(176)		Somewhat remote(176)		Remote(216)		
	n	% (95%CI)	n	% (95%CI)	n	% (95%CI)	p
DM	11	6.3(3.4–10.6)	11	6.3(3.4–10.6)	4	1.9(0.6–4.3)	0.052
HTN	57	32.4(25.8–39.5)	44	25.3(19.3–32.1)	62	28.7(23–35)	0.3
LDL mg/dl	14	8(4.6–12.6)	9	5.1(2.6–9.1)	5	2.3(0.9–5)	0.04
HDL	141	80.1(73.8–85.5)	144	81.8(75.6–87)	183	84.7(79.5–89)	0.5
Total cholesterol mg/dl	17	9.7(6–14.7)	10	5.7(3–9.8)	6	2.8(1.2–5.6)	0.02
Triglycerides	43	24.4(18.5–31.2)	11	26.7(20.6–33.6)	4	18.1(13.4–23.6)	0.1
VLDL mg/dl	10	5.7(3–9.8)	44	11.9(7.8–17.3)	62	5.1(2.7–8.6)	0.02
Dyslipidemia	151	85.8(80.1–90.4)	9	84.1(78.2–88.9)	5	87(82.1–91)	0.7

DM = Diabetes Mellitus, HTN = Hypertension (high blood pressure), LDL = low-density lipoprotein, HDL = High-density lipoprotein, VLDL = very low density lipoprotein.

The prevalence of diabetes was 4.6% (95%CI 3.1–6.5), while hypertension 28.8% (95%CI 25.2–32.6), and dyslipidemia 85.7% (95% CI 82.7–88.4) were much higher. Specifically, HDL abnormality was most prevalent 82.4% (95% CI 79.1–85.4), followed by triglycerides 22.7% (95% CI 19.5–26.4), LDL 4.9% (95% CI 3.4–6.9), VLDL 7.4% (95% CI 5.6–10), and total cholesterol 5.8% (95% CI 4.1–8). Interestingly, diabetes prevalence was significantly higher in the non-Tribal area. However, despite higher levels of modifiable risk factors and lower visceral fat levels among Tribal participants, there were no significant differences in lipid abnormalities or hypertension between Tribal and non-Tribal participants within the tribal area. (see Table 5).

**Table 5:** Comparison of NCD f of the study participants among tribal and non-tribal participants in the tribal area.

	Non-tribal(233)		tribal (335)		Total(568)		
	n	%(95%CI)	n	%(95%CI)	n	%(95%CI)	p
DM	18	7.7(4.8–11.7)	8	2.4(1.1–4.5)	26	4.6(3.1–6.5)	0.003
HTN	70	30(24.4–36.1)	93	27.9(23.3–32.9)	163	28.8(25.2–32.6)	0.6
LDL mg/dl Increased (>130mg/dl)	16	6.9(4.1–10.7)	12	3.6(2–6)	28	4.9(3.4–6.9)	0.08
HDL Low(M<40, F<50)	202	86.7(81.9–90.6)	266	79.4(74.8–83.5)	468	82.4(79.1–85.4)	0.03
TC Increased (>200mg/dl)	15	6.4(3.8–10.1)	18	5.4(3.3–8.2)	33	5.8(4.1–8)	0.6
TGA High (>150mg/dl)	53	22.7(17.7–28.4)	76	22.7(18.4–27.4)	129	22.7(19.4–26.3)	0.98
VLDL >50 mg/dl	22	9.4(6.2–13.7)	20	6(3.8–8.9)	42	7.4(5.5–9.8)	0.1
Dyslipidemia	208	89.3(84.8–92.8)	279	83.3(79–87)	487	85.7(82.7–88.4)	0.045

DM = Diabetes Mellitus, HTN = Hypertension (high blood pressure), LDL = low-density lipoprotein, HDL = High-density lipoprotein, TC = Total cholesterol, TGA = Triglycerides, VLDL = very low density lipoprotein.

### Association of diabetes and hypertension among study participants

All the significant variables at univariate analysis by p<0.1 were included in the multiple logistic regression. In the univariate analysis (irrespective of tribal status),we found that increasing age, increasing waist circumference, and being remote were independently associated with diabetes. Among the same group, only increasing age, higher frequency of red meat consumption, and higher waist circumference were significantly associated with hypertension in the multivariate logistic regression analysis (S1 Table).

Among tribal participants, only high waist circumference was independently associated with diabetes, while increasing age, increasing waist circumference, and increasing wealth index were independent predictors of hypertension in the multivariate logistic regression analysis (S2 Table).

In the tribal area, there was a statistically significant difference in HDL cholesterol levels between tribal and non-tribal participants. HDL cholesterol levels were slightly lower in the tribal area by 7%. On the other hand, diabetes prevalence was significantly higher in the non-tribal area compared to the tribal area.

Regarding lifestyle factors, tribal participants had significantly higher rates of smoking, smokeless tobacco use, and underweight, and lower rates of obesity compared to non-tribal participants. Additionally, non-tribal participants had a more frequent consumption of meat, chicken, processed food, and aerated drinks, and a less frequent consumption of vegetables compared to tribal participants.

In the study, 568 participants had their blood pressure measured, and the results showed that 41.1% had normal blood pressure, 35.9% were in pre-hypertension, 16.5% had stage 1 hypertension, and 6.4% had stage 2 hypertension. Out of all the participants, 23% had uncontrolled blood pressure, and there were more unknown hypertensive participants (111/163) compared to known (56/163). Only 3 out of 568tribal participants reported practicing yoga, and 33.8% of participants had recorded their blood pressure within the last year, which is a good proportion considering the remote and rural demographic of the study. Among the 52 known hypertensive participants, only 26 were on medication, and 19.2%, 7.7%, and 15.4% were advised on losing weight, quitting smoking, and regular exercise, respectively. Only 1.9% of those advised were on AYUSH medication for hypertension.

In the study population, there were 568 participants with fasting blood glucose data available. Among them 11 (2%) were in the impaired fasting glucose category and 17 (3%) were in the diabetes category. The known to new diabetes cases ratio was 1.3, indicating that the screening program implementation was in line with NPCDS guidelines. Out of the 15 participants who knew they had diabetes 11(73%) were taking oral hypoglycaemic agents and 8/15 (53.3%) were taking insulin. Only 37/568 (6.5%) were tested for blood sugar in last one year. Only half of the participants (7/15) were advised on a modified diet for diabetes. Approximately one-third (6/15) reported receiving advice for weight reduction, and a similar proportion reported receiving advice about regular exercise. None of the participants with diabetes reported taking AYUSH medicines.

## Discussion

Through this study, we report striking differences in the prevalence of metabolic disorders among tribal and non-tribal populations in a remote area of southern India. The prevalence of hypertension, dyslipidemia, and diabetes was alarmingly high with the latter being significantly higher in the non-tribal population. Additionally, significant differences were observed in diabetes, LDL abnormality, TGA abnormality, VLDL abnormality, and TC abnormality across remoteness. However, no significant differences were noted in hypertension, dyslipidemia, and HDL abnormality across remoteness. Among the forest dwelling population, there were significant differences in diabetes and HDL abnormality, but no significant differences in hypertension, dyslipidemia, LDL abnormality, TGA abnormality, TC abnormality, and VLDL abnormality between tribal and non-tribal participants. These findings underscore the need for tailored interventions to address the growing burden of metabolic disorders in this vulnerable population.

Non-tribal participants had a higher prevalence of risk factors such as high waist circumference (41.3% vs. 19.3%), underweight (33.1% vs. 49.6%), smoking (25% vs. 40.6%), and use of smokeless tobacco (9.2% vs. 18.7%), as well as more frequent consumption of fried food(49.6% vs. 57.6%), processed food(32.8% vs.34.5%), red meat(36.5% vs. 60.5%), and aerated drinks(30.6% vs. 19.8%), and lower daily vegetable use (59.7% vs. 51.2%) and fruit consumption (31.3% vs. 36.5%) compared totribal participants.

Age and marital status are comparable to general population distribution (census of India), the literacy rate is less than the tribal population of Chamarajanagar (61%), and however, since this is a forest-dwelling population, which has less literacy rate than the rest of population, the study sample is comparable with respect to literacy rate. Other tribal studies reported comparable age, gender distribution and literacy rate [15,31,46–49]. According to the census of India 2011, there are 1020 females for 1000 males in chamarajanagar tribal [50]. Even compared to this, the study had an over-representation of females, due to already mentioned reasons.

### Hypertension

The current study found a hypertension prevalence of 28.8%, which is comparable to the general population (28.5%) [51]. However, the prevalence was higher than that reported by Madhu et al (20.7%) in the same setting (MM hills), which may be due to the limited sampling to 4 villages, not representative of the entire tribal region [52]. On the other hand, studies conducted in Madhya Pradesh (54.3%), Uttarakhand (43.4%), Kerala (48.3%), Nicobar (50%), and Assam (33%) reported higher prevalence rates than the current study [53–55]. In contrast, multicentric studies by Kshatriya et al (10–13%), HP (Sunil Kumar et al) (10.7%), coastal Maharashtra (Deo et al) (16.7%), Rajasthan (Sachdeva et al) (22.8%), Karnataka (Basavanagoudappa et al) (21.7%), and Gujarat (RR Tiwari, 16.9%) and Systematic review and metanalysis among tribal population (23.7%) reported lower prevalence rates than the current study [28,47,56–59]. Considering the timeline of these studies which were conducted a decade earlier might be the reason for the difference in prevalence. The Himachal Pradesh study is much similar to our study in terms of high altitude. Lakshmaih et al., Meshram et al reported comparable hypertension prevalence as our study [48,60].

The differences in the prevalence of hypertension among different tribal population might be due to timeline, extent of acculturation, location (altitude, forest/plain), type of tribal, built of the tribal, the dietary pattern, alcohol use and tobacco use, physical activity [55,61]. Most of the tribals are physically very active except for the gonda tribal of mandla [31]. The prevalence of hypertension has increased significantly among tribal population mainly due to increased consumption of tobacco, alcohol and sedentary lifestyle and needs urgent action in terms of awareness for preventing activities. Some of the studies showed that smoking physical inactivity, alcohol usewere also significant predictors [48,58,61,62]. Gautam Kshatriya et al. reported a 10% prevalence of hypertension; undernutrition was a significant risk factor [56].

Medication adherence in the present study was comparable with other tribal populations [31,63]. However, nearly 50% hypertensive and diabetics knew their status, which is better than other tribal populations which reported in the range of 10–24% [31,55,62,63].

### Diabetes

The current study found a prevalence of 4.6% for diabetes and 1.94% for impaired fasting glucose (IFG). The new to known cases ratio was 1:1.4, suggesting effective screening by the NPCDS program. However, among tribal participants, the prevalence was lower at 2.9% for diabetes and 1.8% for IFG. The multicenter diabetes study on the general population reported a higher prevalence of diabetes at 7.3% [64]. Hence,tribal diabetes prevalence is lower than the general population. Compared to the current study, Varhlun chhungi et al (Liangmai tribe,2010–12, 4.9%), Sachidev et al (2009, 5.2%), Varhlunchhungi et al (mizotribe,2010–12, 32.8%), Kappor et al (MP, 2010, 7.9%), Negi et al (HP. 2014,6.9%), Sarkar et al (2002, Sikkim-Bhutan border, 10.1%), and Deo et al (2014, 11.6%, Maharashtra) reported much higher prevalence of diabetes [27,28,30,63,65]. Also, Sachdev (2012, Rajasthan, 3.9%), Sathiyanarayan et al (Jawadu tribe of Tamil Nadu, 2018, 3.78%), D kappor et al (2008–10, MP, 4.1%) and Madhu et al (Chamarajanagar, 2019, 2.9%) reported similar prevalence of diabetes [29,52,66,67]. The prevalence of Impaired fasting glucose was very less in the present study, 1.8%. A similar lower prevalence is seen (1.8% Toto tribal of West Bengal), Sarkar S et al (2.4%) [27]. However, Sachdev et al. (6.6%) and Satyanarayana reported (9.4%) reported higher IFG prevalence [13,66].

The higher prevalence of diabetes and pre diabetes could be due to self-selection bias, as sampling method within these population would be difficult as evidenced by non-reporting of sampling methodology by studies and also due to logistic difficulty on fasting samples and acceptability [13,68]. Diabetes was associated independently with abdominal obesity in the current study as reported by other tribal studies. Most of the tribal people were leading a physically active lifestyle and as the transport was very limited in most villages, they had to walk to every place for their occupation or any other work. So comparatively, obesity was less among these people in the remote areas and the prevalence of diabetes was also less compared to the general population [17,69].

Dyslipidemia overall is comparable to the rural general population as reported by the INDIAB multicentre study (Also, this was done almost a decade back, during which significant changes in life style has occured); however, it is mainly contributed by the low HDL and high TGA in the present study. However, LDL and total cholesterol were high even in the general rural population [64]. The high proportion of low HDL is to be evaluated. Further, this is despite high level of physical activity in these forest dwelling population.

Other studies in the tribal area like Ganie et al at Kashmir tribal population, Varhulun et al. (2019) from Manipur, reported a similar prevalence of dyslipidemia[28,70]. The present study had a high prevalence of dyslipidemia compared to AK Bharadwaj et al. and Sarkar et al. However, these were done in 2013 and 2005 [27,71].

Tribals are highly endogamous and marry within the closed community [72]. In the current study, endogamy among tribals might explain the variation in the pattern and prevalence of non-communicable diseases (NCDs), which are known to be influenced by race [60]. The prevalence of alcohol use was relatively lower in the current study compared to other Tribal area [14,46,53,55,62,73]. Negi et al., Madhu et al., Vishnu et al. and D Kapoor et al., also reported similar lower levels of smoking and alcohol [52,62]. However, the prevalence of obesity and overweight, including abdominal obesity, was found to be higher in the current study compared to other studies [15,28,31,52,74,75].

In the current study, malnutrition characterized by both under-nutrition and obesity was observed among tribal participants, indicating a dual burden of cardiovascular disease. This is consistent with several other studies that reported a high prevalence of undernutrition, indicating a double burden to the tribal population. These findings suggest that the tribal population’s nutritional status needs to be addressed through targeted interventions, as seen in urban and peri-urban communities with more extensive data [14,60,65,75]. Though majority studies reported a similarly high level of physical activity, surprisingly, even though the hilly area, only 23% heavy activities, similarly Vishnu et al. also reported only 55% doing moderate to heavy activities, which is way less than most of the tribal studies, which report around 80% [29,31,46,57]. Vegetable usage is also very less like the present study. Deo et al. also reported that only 32% used vegetables at least 3 days a week [14]. Current study findings of insufficient fruit and vegetable intake are comparable with the NNMS, pan India survey among the general population on NCD risk factors[51]. The nutrition transition among both tribal and rural populations has resulted in a shift away from indigenous traditional diets to increased consumption of processed foods. Jerath et al. reported that despite the availability of diverse natural foods, household access to them is poor [9]. The reasons for this disparity include rapid nutrition transition with greater market access, the high cost of obtaining natural food sources, forest conservation laws, and the gradual erosion of transgenerational traditional ecological knowledge (TEK) [9].

The phenomenon of globalization-driven nutritional transition has led to significant changes in food habits, particularly affecting the poor who are vulnerable to food insecurity. Hawkes (2006) highlighted the cultural convergence towards low-quality diets, such as inexpensive vegetable oil and trans-fat [76]. In the NNMB (2009)report, tribal women have been observed with an elevated intake of fats, oils, sugar at an overall level, along with a fall in intake of vegetables [77]. The report further suggests an essential ‘food gap’ among the Indian tribal due to the highly pronounced dietary energy and protein inadequacy.

The study has identified the non-reachability of the health system in remote tribal area represented by significant number of undiagnosed cases of diabetes, hypertension and dyslipidaemia, though better than other studies, indicating the need for different strategy of NP-NCD to make sure that they reach the unreachable [69]. The study also has identified high level of NCD risk factors in terms of alcohol use, smoking, more than expected levels of fried food, processed food and inadequate use of fruits and vegetables. The places where internet and electricity have not reached, but these high risk behaviour have reached, should making us to rethink of strategies to educate and empower them to rid of these follow healthy behaviours [78]. Empowerment is specially needed with regard to growing vegetables and with within the rules of permissions for the same as forest act, prohibits them to do so. In contrary to most people expectation, that there must be plenty of fruits available in hilly region, the animals like monkey and elephants do not facilitate residents to consume, as they either eat away or spoil the fruits.

Education on alcohol and smoking de-addiction also need to be focussed in these forest population by the NP-NCD program. Also, being forest area Frequent interaction with tourists coming there and their visits to outside world has resulted in acculturation in relation addictions and eating habits, leading to use of processed food, eating outside the home more frequently, use of aerated drinks and use of sweet drinks.

Also, these unhealthy dietary habits like more frequent use of red meat, chicken and fried foods resulting in high levels of inflammatory markers, which result in higher risk of hypertension and diabetes. The response rate was better than some other studies in the tribal area [27]. The reasons for non-response were fasting for 12 hrs and reasons like illness (minor illness), especially want to smoke early in the morning, temporary migration. Similar reasons were reported by Sarkar et al [27]. There are no age and gender distribution of the tribal population in the public domain. Hence the comparison was not possible. However, several other tribal NCD studies reported a similarly high proportion of young and middle-aged participants [58], higher female and lower literacy rate, higher kaccha houses as the present study [49,55].

### Strengths

The response rate was better than some other studies in the tribal area. The reasons for non-response were fasting for 12 hrs and reasons like illness (minor illness), especially want to smoke early in the morning, temporary migration. Similar reasons were reported by Sarkar et al [27]. There are no age and gender distribution of the tribal population in the public domain. Hence the comparison was not possible. This study is significant as it assessed the prevalence of diabetes, hypertension, and dyslipidemia in a single tribal study using the STEPS questionnaire. It also provided clear demarcation of tribal and non-tribal populations based on caste within the tribal population, allowing for differentiation of possible genetic origins of non-communicable diseases. Moreover, the use of specific definitions to identify tribal within the population residing in the tribal area facilitated the analysis of differences between the two categories. Additional analyis is available in S2 Text

### Limitation

In the present study, the Kish method could not be implemented completely, resulting in an over-representation of females, although the ST population in the Chamarajanagar district is 1.027 males for every female. Moreover, OGTT could not be performed for all participants, and only those with higher FBS underwent the test, which may have led to an underestimation of the prevalence of diabetes to some extent. Additionally, social desirability bias could have affected the reporting of alcohol use by participants.

## Conclusions and recommendations

The present study highlights that the prevalence of hypertension and dyslipidemia is high among tribal populations, which is comparable to the general population and higher than some rural areas. The study also reveals that the dietary habits of tribal populations are shifting towards unhealthy urban habits with insufficient intake of fruits and vegetables as per WHO standards. Moreover, smoking and alcohol use are prevalent among tribal populations. However, the study finds that a considerable proportion of tribalpopulations still engage in higher levels of physical activity. The study recommends that special focus on NCD care in the tribal community is necessary through mobile NCD care units provided by NPCDS. Additionally, tribal should be cautious regarding the rapid acculturation of unhealthy lifestyle habits. It is imperative to take urgent action to improve the NCD care in the tribal area to reduce the burden of NCDs in this vulnerable population.

Contrary to the popular belief that NCDs are less prevalent in tribal areas, our study found a higher prevalence of hypertension and dyslipidemia, comparable to rural areas, and a comparable prevalence of diabetes (except for very remote areas). Therefore, it is crucial to focus on NCD surveillance in tribal areas similar to other rural/urban settings. Our study recommends setting up NCD surveillance in tribal areas due to the high prevalence of hypertension (comparable to the general population) and other NCD risk factors, along with the need for genetic exploration of hypertension and dyslipidemia. Moreover, the study highlights the poor NCD care in terms of drugs and non-pharmacological care. The diabetes prevalence was lowest in remote areas, suggesting the need to restrict the availability of processed and fried foods and facilitate education to the community on a healthy diet and quitting tobacco and alcohol through de-addiction services.

## Supporting information

S1 Table Multivariate logistic regression for diabetes and hypertension among all study subjects. (DOCX)

S2 Table Multivariate logistic regression for diabetes and hypertension among tribal subjects. (DOCX)

S1 Text Metabolic Equivalents calculation. (DOCX)

S2 Text Additional analysis. (DOCX)

## References

[1] World Health Organization. Non communicable diseases [Internet]. Noncommunicable diseases. 2023 [cited 2024 Mar 3]. Available from: https://www.who.int/news-room/fact-sheets/detail/noncommunicable-diseases.

[2] International Diabetes Federation. Diabetes around the world in 2021 [Internet]. IDF Diabetes Atlas. 2021 [cited 2023 Dec 10]. Available from: https://diabetesatlas.org/.

[3] Kearney PM, Whelton M, Reynolds K, Muntner P, Whelton PK, He J. Global burden of hypertension: Analysis of worldwide data. Lancet. 2005 Jan;365(9455):217–23. doi: 10.1016/S0140-6736(05)17741-1 15652604

[4] Indian Council of Medical Research, Public Health Foundation of India, Metrics I for H, Evaluation. India: Health of the Nation’s States- The India State-level Disease Burden Initiative. New Delhi; 2017.

[5] Prabhakaran D, Jeemon P, Sharma M, Roth GA, Johnson C, Harikrishnan S, et al. The changing patterns of cardiovascular diseases and their risk factors in the states of India: the Global Burden of Disease Study 1990–2016. Lancet Glob Health. 2018 Dec;6(12):e1339–51. doi: 10.1016/S2214-109X(18)30407-8 30219317 PMC6227386

[6] Kumar A, Bhatia M, Goel P, Jain R. Diabetes in Tribes of India: A literature review. J Soc Health Diabetes. 2016 Jun;04(01):41–3.

[7] MoTA. Statistical Profile of Scheduled Tribes in India [Internet]. 2013. p. 1–448. Available from: www.tribal.nic.in

[8] Bisai S, Saha KB, Sharma RK, Muniyandi M SN. An overview of tribal population in India. Tribal Health Bulletin. 2014 p. 1–126.

[9] Ghosh-Jerath S, Kapoor R, Bandhu A, Singh A, Downs S, Fanzo J. Indigenous Foods to Address Malnutrition: An Inquiry into the Diets and Nutritional Status of Women in the Indigenous Community of Munda Tribes of Jharkhand, India. Curr Dev Nutr. 2022 Sep 1;6(9):nzac102. doi: 10.1093/cdn/nzac102 36110104 PMC9470035

[10] Chapter Bose P. 4—Forest foods for tribals in selected regions of India and their sustainability. In: Prakash J, Waisundara V, Prakash V, editors. Nutritional and Health Aspects of Food in South Asian Countries [Internet]. Academic Press; 2020 [cited 2023 Jun 26]. p. 51–9. (Nutritional & Health Aspect-Traditional&Ethnic Food). Available from: https://www.sciencedirect.com/science/article/pii/B9780128200117000058.

[11] Rizwan SA, Kumar R, Singh AK, Kusuma YS, Yadav K, Pandav CS. Prevalence of Hypertension in Indian Tribes: A Systematic Review and Meta-Analysis of Observational Studies. PLoS ONE [Internet]. 2014 May;9(5). Available from: https://www.ncbi.nlm.nih.gov/pmc/articles/PMC4010404/. doi: 10.1371/journal.pone.0095896 24797244 PMC4010404

[12] Upadhyay RP, Misra P, Chellaiyan VG, Das TK, Adhikary M, Chinnakali P, et al. Burden of diabetes mellitus and prediabetes in tribal population of India: A systematic review. Diabetes Res Clin Pract. 2013;102(1):1–7. doi: 10.1016/j.diabres.2013.06.011 23876547

[13] Sachdev B. Community based study on incidence of type 2 diabetes and hypertension among nomad tribal population of Rajasthan, India. Int J Sci Nat. 2011;2(2):296–301.

[14] Deo MG, Pawar PV, Kanetkar SR, Kakade SV. Prevalence and risk factors of hypertension and diabetes in the Katkari tribe of coastal Maharashtra. J Postgrad Med. 2017 Jun;63(2):106–13. doi: 10.4103/0022-3859.194204 27853041 PMC5414420

[15] Kandpal V, Sachdeva MP, Saraswathy KN. An assessment study of CVD related risk factors in a tribal population of India. BMC Public Health. 2016 May 25;16(1):434. 27225632 10.1186/s12889-016-3106-x PMC4880982

[16] Majgi S, Suresh H, Nuggehalli Srinivas P, Ahmed M. Prevalence of Hypertension Among Tribal Population in India: A Systematic Review and Meta-Analysis. Natl J Community Med. 2023 May 1;14(05):276–83.

[17] Tripathi N, Kishore J, Chaitanya V, Kumar P, Babu BV. Prevalence of Diabetes Mellitus in Indian Tribal Population: A Systematic Review and Meta-analysis. Ann Community Health. 2021 Jan 1;8(4):169–77. 10.1080/13557858.2022.2067836 35469488

[18] Hazarika CR, Babu BV. Prevalence of diabetes mellitus in Indian tribal population: a systematic review and meta-analysis. Ethn Health. 2023 May;28(4):544–61. doi: 10.1080/13557858.2022.2067836 35469488

[19] Srinivas PN, Seshadri T, Velho N, Babu GR, Madegowda C, Channa Basappa Y, et al. Towards Health Equity and Transformative Action on tribal health (THETA) study to describe, explain and act on tribal health inequities in India: A health systems research study protocol [version 1; peer review: 2 approved]. Wellcome Open Res. 2019;4(202). 10.12688/wellcomeopenres.15549.1 PMC7076281 32211518

[20] Ministry of Tribal Affairs, Government of India. State/Union Territory-wise list of Scheduled Tribes in India [Internet]. 2022. Available from: https://tribal.nic.in/ST/LatestListofScheduledtribes.pdf.

[21] Government of Karnataka. Human Development: Performance of Districts, Taluks and Urban Local Bodies in Karnataka, 2014-Snapshot. 2014. p. 89–94.

[22] Rowold DJ, Chennakrishnaiah S, Gayden T, Luis JR, Alfonso-Sanchez MA, Bukhari A, et al. The Y-chromosome of the Soliga, an ancient forest-dwelling tribe of South India. Gene. 2020 Dec;763:100026. 10.1016/j.gene.2019.100026 34493361

[23] QGIS Development Team. QGIS Geographic Information System [Internet]. 2018. Available from: https://qgis.org/en/site/.

[24] Satpathi AD Sayantani. Forests, People, and Their Hopes: PESA and FRA and Overview. In: Tribal Development Report. Routledge India; 2022.

[25] Thresia CU, Srinivas PN, Mohindra KS, Jagadeesan CK. The Health of Indigenous Populations in South Asia: A Critical Review in a Critical Time. Int J Health Serv Plan Adm Eval. 2022 Jan;52(1):61–72. 10.1177/0020731420946588 PMC7611999 32787539

[26] Sudarshan H, Prashanth NS. Good governance in health care: the Karnataka experience. The Lancet. 2011 Mar 5;377(9768):790–2. 10.1016/S0140-6736(10)62041-7 21227488

[27] Sarkar S, Das M, Mukhopadhyay B, Chakrabarti CS, Majumder PP. High prevalence of metabolic syndrome and its correlates in two tribal populations of India and the impact of urbanization. Indian J Med Res. 2006 May;123(5):679–86. 16873911

[28] Chhungi V, Ningombam SS, Yadav S, Singh HS, Devi NK, Chandel S, et al. Prevalence of cardiovascular risk factors among tribal and non-tribal populations with East Asian Ancestry from North East India. Am J Hum Biol Off J Hum Biol Counc. 2019 Sep;31(5):e23263. doi: 10.1002/ajhb.23263 31197927

[29] Kapoor D, Bhardwaj AK, Kumar D, Raina SK. Prevalence of Diabetes Mellitus and Its Risk Factors among Permanently Settled Tribal Individuals in Tribal and Urban Areas in Northern State of Sub-Himalayan Region of India. Int J Chronic Dis. 2014;2014:380597. doi: 10.1155/2014/380597 26464856 PMC4590924

[30] Deo MG, Pawar PV, Kanetkar SR, Kakade SV. Multicentric study on prevalence and risk factors for hypertension and diabetes in tribal communities in Western and Northern Maharashtra. J Postgrad Med. 2018;64(1):23–34. doi: 10.4103/jpgm.JPGM_245_17 29386415 PMC5820811

[31] Gupta VK, Rai N, Toppo NA, Kasar PK, Nema P. An epidemiological study of prevalence of hypertension and its risk factors among non migratory tribal population of Mawai block of Mandla district of central India. Int J Community Med Public Health. 2018;5(March):957–62.

[32] Dean A. OpenEpi: open source epidemiologic statistics for public health. HttpwwwOpenEpiCom. 2007;

[33] Kish L. A Procedure for Objective Respondent Selection within the Household. J Am Stat Assoc. 1949;44(247):380–7.

[34] World Health Organization. WHO STEPS Instrument for Chronic Disease [Internet]. 2009 p. 12. Available from: http://www.who.int/chp/steps/STEPS_Instrument_v2.1.pdf.

[35] Rutstein SO, Johnson K. The DHS wealth index [Internet]. Calverton, Maryland, USA: ORC Macro; 2004. (DHS Comparative Reports No. 6.). Available from: http://dhsprogram.com/pubs/pdf/CR6/CR6.pdf.

[36] National Institute of Medical Statistics, Indian Council of Medical Research (ICMR). IDSP Non-Communicable Disease Risk Factors Survey, Phase-I States of India,2007–08. New Delhi, India: National Institute of Medical Statistics and Division of Non-Communicable Diseases, Indian Council of Medical Research,; 2009.

[37] Sjostrom M, Ainsworth BE, Bauman A, Bull FC, Hamilton-Craig CR, Sallis JF. Guidelines for data processing analysis of the International Physical Activity Questionnaire (IPAQ)—Short and long forms. In 2005. Available from: https://api.semanticscholar.org/CorpusID:79242415.

[38] science N institute of medical. Integrated disease surveillance project. Ministry of health and family welfare. New Delhi; 2007 p. 1–142.

[39] WHO Expert Consultation. Appropriate body-mass index for Asian populations and its implications for policy and intervention strategies. Lancet Lond Engl. 2004 Jan 10;363(9403):157–63. doi: 10.1016/S0140-6736(03)15268-3 14726171

[40] World Health Organization. Definition, Diagnosis and Classification of Diabetes Mellitus. Part 1: Diagnosis and Classification of Diabetes Mellitus. Geneva, WHO. 1999.

[41] NCEP. Third Report of the National Cholesterol Education Program (NCEP) Expert Panel on [Internet]. 01–3670. 2001 p. 40. Available from: http://www.nhlbi.nih.gov/files/docs/guidelines/atp3xsum.pdf.

[42] World Health Organization. The WHO STEPwise approach to noncommunicable disease risk factor surveillance. World Heal Organ. 2017;36:1–474.

[43] James PA, Oparil S, Carter BL, Cushman WC, Dennison-Himmelfarb C, Handler J, et al. 2014 Evidence-Based Guideline for the Management of High Blood Pressure in Adults Report From the Panel Members Appointed to the Eighth Joint National Committee (JNC 8) Clinical Review & Education Special Communication 507. JAMA. 2014;311(5):507–20. 24352797 10.1001/jama.2013.284427

[44] Dyslipidemia—an overview | ScienceDirect Topics [Internet]. [cited 2024 Feb 25]. Available from: https://www.sciencedirect.com/topics/medicine-and-dentistry/dyslipidemia.

[45] Response to correspondence article on the… | Wellcome Open Research [Internet]. [cited 2023 Jul 31]. Available from: https://wellcomeopenresearch.org/articles/8-155/v1.

[46] Sajeev P, Soman B. Prevalence of noncommunicable disease risk factors among the Kani tribe in Thiruvananthapuram district, Kerala. Indian Heart J. 2018;70(5):598–603. doi: 10.1016/j.ihj.2018.01.022 30392494 PMC6204451

[47] Chakma T, Kavishwar A, Sharma RK, Rao PV. High prevalence of hypertension and its selected risk factors among adult tribal population in Central India. Pathog Glob Health. 2017 Oct;111(7):343–50. doi: 10.1080/20477724.2017.1396411 29139339 PMC5694887

[48] Laxmaiah A, Meshram II, Arlappa N, Balakrishna N, Mallikharjuna Rao K, Reddy CG, et al. Socio-economic & demographic determinants of hypertension & knowledge, practices & risk behaviour of tribals in India. Indian J Med Res [Internet]. 2015; Available from: https://www.ncbi.nlm.nih.gov/pmc/articles/PMC4510771/. 10.4103/0971-5916.159592 PMC4510771 26139790

[49] Ganie MA, Habib A, Ali SA, Rashid A, Rashid R, Fazili A. Cross sectional study on Kashmiri tribal population: Their demo-economic status and behavioural risk factors. J Fam Med Prim Care. 2020 Sep;9(9):4929–35. doi: 10.4103/jfmpc.jfmpc_745_20 33209824 PMC7652166

[50] Census of India. Population composition. Census of India. 2011.

[51] Id PM, Kulothungan V, Leburu S. PLOS ONE National noncommunicable disease monitoring survey (NNMS) in India: Estimating risk factor prevalence in adult population. 2021;1–17. 10.1371/journal.pone.0246712 PMC7924800 33651825

[52] Madhu B, Prathyusha K, Prakruthi P, Srinath KM. Comparison of prevalence of life style risk factors and 10 year risk of CVD event among rural and tribal population of Kollegal Taluk, Chamrajanagar district, South India. Diabetes Metab Syndr. 2019 Oct;13(5):2961–6. doi: 10.1016/j.dsx.2019.07.056 31425964

[53] Manimunda SP, Sugunan AP, Benegal V, Balakrishna N, Rao MV, Pesala KS. Association of hypertension with risk factors & hypertension related behaviour among the aboriginal Nicobarese tribe living in Car Nicobar Island, India. Indian J Med Res. 2011 Mar;133(3):287–93. 21441682 PMC3103153

[54] Hazarika N, Narain K, Biswas D, Kalita H, Mahanta J. Hypertension in the native rural population of Assam. Natl Med J India. 2003 Nov;17:300–4. 15736549

[55] Raina SK, Chander V, Prasher CL, Raina S. Prevalence of Hypertension in a Tribal Land Locked Population at High Altitude [Internet]. Scientifica. 2016. Available from: https://www.hindawi.com/journals/scientifica/2016/3589720/. 10.1155/2016/3589720 PMC4773562 26989560

[56] Kshatriya GK, Acharya SK. Prevalence and risks of hypertension among Indian tribes and its status among the lean and underweight individuals. Diabetes Metab Syndr Clin Res Rev. 2019 Mar;13(2):1105–15. doi: 10.1016/j.dsx.2019.01.028 31336452

[57] Hathur B, Basavegowda M, Kulkarni P, Ashok NC. Metabolic syndrome among diabetics and pre-diabetics of Jenu Kuruba tribe in Mysore district (JKDHS-2)—An evidence of metabolic abnormalities leading to increase in CVD’s among Jenu Kuruba tribal population. Diabetes Metab Syndr Clin Res Rev. 2015. doi: 10.1016/j.dsx.2015.08.004 26359305

[58] Tiwari RR. Hypertension and epidemiological factors among tribal labour population in Gujarat. Indian J Public Health. 2008 Sep;52(3):144–6. 19189836

[59] Hazarika CR, Babu BV. Prevalence of Hypertension in Indian Tribal Population: a Systematic Review and Meta-analysis. J Racial Ethn Health Disparities. 2023 Feb 8. doi: 10.1007/s40615-023-01532-6 36752902

[60] Meshram I. I. L. Prevalence of Hypertension and Its Correlates among Adult Tribal Population (≥20 Years) of Maharashtra State, India. -. Int J Health Sci Res IJHSR. 2014.

[61] Kusuma YS, Babu BV, Naidu JM. Prevalence of hypertension in some cross-cultural populations of Visakhapatnam district, South India. Ethn Dis. 2004;14(2):250–9. 15132211

[62] Meshram I, Arlappa N, Balkrishna N, Rao K, Laxmaiah A, Brahmam G. Prevalence of hypertension, its correlates and awareness among adult tribal population of Kerala state, India. J Postgrad Med. 2012;54(4):255. doi: 10.4103/0022-3859.105444 23298919

[63] Negi PC, Chauhan R, Rana V, Vidyasagar, Lal K. Epidemiological study of non-communicable diseases (NCD) risk factors in tribal district of Kinnaur, HP: A cross-sectional study. Indian Heart J. 2016 Sep;68(5):655–62. doi: 10.1016/j.ihj.2016.03.002 27773404 PMC5079134

[64] Anjana RM, Deepa M, Pradeepa R, Mahanta J, Narain K, Das HK, et al. Prevalence of diabetes and prediabetes in 15 states of India: results from the ICMR-INDIAB population-based cross-sectional study. Lancet Diabetes Endocrinol. 2017 Aug;5(8):585–96. doi: 10.1016/S2213-8587(17)30174-2 28601585

[65] Kapoor S, Tyagi R, Saluja K, Chaturvedi A, Kapoor A. Emerging health threats among a primitive tribal group of Central India. J Public Health Epidemiol. 2010 May;2:13–9.

[66] Sathiyanarayanan S, Muthunarayanan L, Devaparthasarathy TA. Changing Perspectives in Tribal Health: Rising Prevalence of Lifestyle Diseases among Tribal Population in India. Indian J Community Med Off Publ Indian Assoc Prev Soc Med. 2019;44(4):342–6. doi: 10.4103/ijcm.IJCM_40_19 31802797 PMC6881886

[67] Sachdev B. Screening of Type 2 Diabetes Mellitus and Its Associated Risk Factors among Select Tribes of Rajasthan. Int J Health Sci Res [Internet]. 2012 Jan;2. Available from: /paper/Screening-of-Type-2-Diabetes-Mellitus-and-Its-Risk-Sachdev/b1975449bcf1128ed8070727ccf63dac5cefd42e

[68] Sathiyanarayanan S, Muthunarayanan L, Devaparthasarathy T. Changing perspectives in tribal health: Rising prevalence of lifestyle diseases among tribal population in India. Indian J Community Med. 2019. doi: 10.4103/ijcm.IJCM_40_19 31802797 PMC6881886

[69] Shriraam V, Mahadevan S, Arumugam P. Prevalence and Risk Factors of Diabetes, Hypertension and Other Non-Communicable Diseases in a Tribal Population in South India. Indian J Endocrinol Metab. 2021;25(4):313–9. doi: 10.4103/ijem.ijem_298_21 35136738 PMC8793947

[70] Ganie MA, Sahar T, Rashid A, Baba MS, Ahmad N, Bhat H, et al. Prevalence of diabetes and prediabetes in tribal population of Kashmir: Lessons for the future. Diabetes Res Clin Pract. 2020 Nov;169:108457. doi: 10.1016/j.diabres.2020.108457 32979420

[71] Bhardwaj AKAK, Kumar D, Raina SKSK, Bansal P, Bhushan S, Chander V. Community based assessment of biochemical risk factors for cardiovascular diseases in rural and tribal area of Himalayan region, India. Biochem Res Int. 2013. doi: 10.1155/2013/696845 24455263 PMC3881340

[72] Roy S, Hegde HV, Bhattacharya D, Upadhya V, Kholkute SD. Tribes in Karnataka: Status of health research. Indian J Med Res. 2015 May;141(5):673–87. doi: 10.4103/0971-5916.159586 26139788 PMC4510769

[73] Misra PJ, Mini GK, Thankappan KR. Risk factor profile for non-communicable diseases among mishing tribes in Assam, India: Results from a WHO STEPs survey. Indian J Med Res. 2014;140(September):370–8. 25366204 PMC4248383

[74] Negi PC, Chauhan R, Rana V, Vidyasagar, Lal K. Epidemiological study of non-communicable diseases (NCD) risk factors in tribal district of Kinnaur, HP: A cross-sectional study. Indian Heart J. 2016 Sep 1;68(5):655–62. 27773404 10.1016/j.ihj.2016.03.002 PMC5079134

[75] Diet Sachdev B. and lifestyle: its association with cholesterol levels among Nomad tribal populations of Rajasthan. Int J Med Biomed Res. 2012 Jan 1;1:124–30.

[76] Hawkes C. Uneven dietary development: linking the policies and processes of globalization with the nutrition transition, obesity and diet-related chronic diseases. Glob Health. 2006 Mar 28;2(1):4. doi: 10.1186/1744-8603-2-4 16569239 PMC1440852

[77] Bureau NNM. NATIONAL NUTRITION MONITORING BUREAU (NNMB) Technical Report No. 25. Natl Nutr Monit Bur NNMB Tech Rep No 25. 2009.

[78] Aklin M, Blankenship B, Nandan V, Urpelainen J. The Great Equalizer: Inequality in Tribal Energy Access and Policies to Address It [Internet]. Rochester, NY; 2021 [cited 2023 Dec 14]. Available from: https://papers.ssrn.com/abstract=3854840.

