# Supplementary material for: Prevalence of dyslipidemia, hypertension and diabetes among tribal and rural population in a south Indian forested region
Source: PLOS Glob Public Health. 2024 May 20;4(5):e0002807. doi: 10.1371/journal.pgph.0002807 (PMC11104681; doi:10.1371/journal.pgph.0002807)
Supplement: S1 Table — (DOCX) [file pgph.0002807.s001.docx]

**S1 Table. Multivariate logistic regression for diabetes and hypertension among all study subjects**

|  | Diabetes | | Hypertension | | |
| --- | --- | --- | --- | --- | --- |
| Factor | OR | p |  | OR | p |
| Tribal | 0.392(0.14-1.1) | .075 | Tribal | 1.361(0.797-2.322) | 0.259 |
| Remoteness | Ref- (non remote) | .096 | Remoteness | Ref- (non remote) | 0.309 |
| Somewhat remote | 0.693(0.257-1.89) | .470 | Somewhat remote | 0.727(0.422-1.254) | 0.252 |
| Remote | 0.246(0.069-0.88) | .031 | Remote | 1.101(0.648-1.869) | 0.722 |
| Age in yrs | Ref- <25 Yrs | .016 | Age in yrs | Ref- <25 Yrs | <.001 |
| 26-40 | 0.489(0.112-2.13) | .341 | 26-40 | 2.887(1.277-6.526) | 0.011 |
| 41-55 | 0.694(0.13-3.7) | .670 | 41-55 | 6.337(2.553-15.73) | <.001 |
| >=56 | 2.977(0.579-15.3) | .191 | >=56 | 17.017(5.472-52.916) | <.001 |
| Gender | 0.386(0.122-1.224) | .106 | Gender | 0.732(0.363-1.475) | 0.383 |
| literacy | 0.798(0.284-2.238) | .668 | MS | Ref- Married | 0.902 |
| Ever smoked | 1.908(0.576-6.322) | .291 | Never married | 0.739(0.246-2.217) | 0.589 |
| Waist Circumference | 4.956(1.75-14.06) | .003 | Separated/ divorced | 1.615(0.116-22.513) | 0.721 |
| Constant | 0.106 | .029 | widowed | 0.861(0.386-1.92) | 0.715 |
|  |  |  | literacy | 0.983(0.618-1.561) | 0.941 |
|  |  |  | Wealth index | Ref-LOWEST | 0.019 |
|  |  |  | SECOND | 1.578(0.763-3.262) | 0.218 |
|  |  |  | MIDDLE | 2.578(1.292-5.143) | 0.007 |
|  |  |  | FOURTH | 3.159(1.528-6.532) | 0.002 |
|  |  |  | HIGHEST | 2.081(0.941-4.604) | 0.07 |
|  |  |  | smoking | 0.894(0.495-1.615) | 0.71 |
|  |  |  | Alcohol use | 1.454(0.699-3.024) | 0.316 |
|  |  |  | **Red meat use** | Ref-Never | 0.048 |
|  |  |  | Rarely | 0.526(0.179-1.541) | 0.241 |
|  |  |  | Sometime | 1.062(0.439-2.573) | 0.894 |
|  |  |  | often | 1.474(0.713-3.047) | 0.295 |
|  |  |  | Always | 3.59(1.147-11.237) | 0.028 |
|  |  |  | Chicken use | Ref-Never | 0.195 |
|  |  |  | Rarely | 2.878(0.696-11.891) | 0.144 |
|  |  |  | Sometime | 0.52(0.203-1.336) | 0.174 |
|  |  |  | often | 0.718(0.363-1.419) | 0.34 |
|  |  |  | Always | 0.771(0.248-2.391) | 0.652 |
|  |  |  | **WC** | 2.488(1.534-4.033) | <.001 |
|  |  |  | Constant | 0.048 | <.001 |
|  | (**Hosmer and Lemeshow Test, p 0.4)** and Nagelkerke R Square=0.28 | |  | **Hosmer and Lemeshow Test, p 0.8)** and Nagelkerke R Square=0.27 | |
