# Supplementary material for: Prevalence of dyslipidemia, hypertension and diabetes among tribal and rural population in a south Indian forested region
Source: PLOS Glob Public Health. 2024 May 20;4(5):e0002807. doi: 10.1371/journal.pgph.0002807 (PMC11104681; doi:10.1371/journal.pgph.0002807)
Supplement: S2 Table — (DOCX) [file pgph.0002807.s002.docx]

**S2 Table. Multivariate logistic regression for diabetes and hypertension among tribal subjects**

|  | Diabetes | | Hypertension | | |
| --- | --- | --- | --- | --- | --- |
| Factor | OR | p |  | OR | p |
| Age category | Ref- <25 yrs | .528 | Remoteness | 1.132(0.81-1.583) | 0.3 |
| 26-40 | 0.322(0.046-2.269) | .255 | Age in yrs | Ref- <25 yrs | <.001 |
| 41-55 | 0(0-.) | .997 | 26-40 | 2.026(0.802-5.117) | .135 |
| >=56 | 1.491(0.166-13.373) | .721 | 41-55 | 4.649(1.703-12.69) | .003 |
| Gender | 0.184(0.033-1.036) | .055 | >=56 | 12.46(3.029-51.252) | <.001 |
| Consumption of sweet drink | Ref- Never | .981 | Gender | 0.857(0.335-2.192) | .747 |
| Rarely | 0(0-.) | .997 | Wealth Index | Ref- LOWEST | .014 |
| sometime | 0(0-.) | .997 | SECOND | 3.046(1.151-8.06) | .025 |
| Often | 1.775(0.313-10.058) | .517 | MIDDLE | 5.368(2.001-14.401) | <.001 |
| always | 0(0-.) | .998 | FOURTH | 4.969(1.675-14.741) | .004 |
| WC | 9.446(1.254-71.152) | .029 | HIGHEST | 2.749(0.706-10.694) | .145 |
| Constant | 0.094(-) | .015 | ever used alcohol | 2.32(0.806-6.672) | .119 |
|  |  |  | Added salt use frequency | Ref- Never | .076 |
|  |  |  | Rarely | 1.035(0.383-2.796) | .945 |
|  |  |  | sometime | 3.751(1.315-10.701) | .013 |
|  |  |  | Often | 0.527(0.108-2.568) | .428 |
|  |  |  | always | 3.616(0.638-20.494) | .146 |
|  |  |  | Consumption of red meat | Ref- Never | .116 |
|  |  |  | Rarely | 0.832(0.185-3.734) | .810 |
|  |  |  | sometime | 1.491(0.439-5.061) | .522 |
|  |  |  | Often | 1.591(0.538-4.704) | .401 |
|  |  |  | always | 7.106(1.432-35.249) | .016 |
|  |  |  | Consumption of chicken | Ref- Never | .319 |
|  |  |  | Rarely | 3.914(0.688-22.259) | .124 |
|  |  |  | sometime | 0.534(0.155-1.846) | .322 |
|  |  |  | Often | 0.901(0.35-2.321) | .830 |
|  |  |  | always | 0.782(0.178-3.432) | .744 |
|  |  |  | WC | 2.94(1.421-6.081) | .003 |
|  |  |  | Constant | 0.02 | 0.01 |
| Hosmer and Lemeshow Test p=.843 | Nagelkerke R Square .351 | | Hosmer lemeshow =0.98 | Nagelkerke R Square .33 | |

On logistic regression, taking age, gender ever used alcohol, red meat frequency, sweet drink frequency and Waist Circumference, resulted only Waist Circumference was independent predictor for diabetes among tribal participants.

In logistic regression, considering all variables p<0.1 in univariate analysis, yielded only increasing age, increasing wealth index, and increasing Waist Circumference independently predicting hypertension in tribal participants.
