# Supplementary material for: Prevalence of dyslipidemia, hypertension and diabetes among tribal and rural population in a south Indian forested region
Source: PLOS Glob Public Health. 2024 May 20;4(5):e0002807. doi: 10.1371/journal.pgph.0002807 (PMC11104681; doi:10.1371/journal.pgph.0002807)
Supplement: S1 Text — (DOCX) [file pgph.0002807.s003.docx]

**Metabolic Equivalents calculation**

MET level for walking = 3.3 METs

MET level for Moderate Intensity = 4.0 METs

MET level for Vigorous Intensity = 8.0 METs

Total MET/Week= walking MET-min/week + moderate intensity MET-min/week + vigorous intensity MET-min/week

Categorical Score- three levels of physical activity are proposed

1. Low: No activity is reported OR some activity is reported but not enough to meet Categories 2 or 3.

2. Moderate: Any combination of walking, moderate-intensity or vigorous intensity activities, achieving a minimum of at least 600 MET-min/week up to 3000 MET- minutes/week

3. High: Any combination of walking, moderate- or vigorous- intensity activities accumulating at least 3000 MET-minutes/week
