# Supplementary material for: Prevalence of dyslipidemia, hypertension and diabetes among tribal and rural population in a south Indian forested region
Source: PLOS Glob Public Health. 2024 May 20;4(5):e0002807. doi: 10.1371/journal.pgph.0002807 (PMC11104681; doi:10.1371/journal.pgph.0002807)
Supplement: S2 Text — (DOCX) [file pgph.0002807.s004.docx]

**S3 Table. Association of diabetes and hypertension with demographic variables (Univariate) among all study subjects**

|  | | DM | | | HTN | | |
| --- | --- | --- | --- | --- | --- | --- | --- |
|  | | n | % | p | n | % | p |
| Tribal | Non Tribal | 18 | 7.7(4.8-11.7) | 0.003 | 70 | 30(24.4-36.1) | 0.6 |
|  | Tribal | 8 | 2.4(1.1-4.5) |  | 93 | 27.9(23.3-32.9) |  |
| Remoteness (SGDI) | Non remote | 11 | 6.3(3.4-10.6) | .052 | 57 | 32.4(25.8-39.5) | 0.3 |
|  | Somewhat remote | 11 | 6.3(3.4-10.6) |  | 44 | 25.3(19.3-32.1) |  |
|  | Remote | 4 | 1.9(0.6-4.3) |  | 62 | 28.7(23-35) |  |
| Age category | <25 | 3 | 2.7(0.8-7) | <0.001 | 9 | 8.2(4.1-14.4) | <0.001 |
|  | 26-40 | 8 | 2.7(1.3-5) |  | 79 | 26.3(21.6-31.5) |  |
|  | 41-55 | 6 | 5.3(2.2-10.5) |  | 49 | 43(34.2-52.2) |  |
|  | >=56 | 9 | 21.4(11.2-35.5) |  | 26 | 61.9(46.8-75.4) |  |
| Gender | Male | 15 | 8.3(4.9-13) | 0.004 | 67 | 37(30.2-44.2) | 0.003 |
|  | Female | 11 | 2.8(1.5-4.9) |  | 96 | 24.9(20.8-29.4) |  |
| Marital status | Married | 19 | 4(2.5-6) | 0.4 | 144 | 30.1(26.1-34.3) | 0.1 |
|  | Never married | 4 | 9.1(3.1-20.2) |  | 5 | 11.9(4.7-24.1) |  |
|  | seperated/Divorced | 0 | 0(.-.) |  | 1 | 33.3(3.9-82.3) |  |
|  | widowed | 3 | 7.1(2.1-17.9) |  | 13 | 31(18.6-45.8) |  |
| literacy | Illiterate | 17 | 5.8(3.5-8.9) | 0.2 | 96 | 32.8(27.6-38.3) | 0.03 |
|  | Literate | 9 | 3.3(1.6-5.9) |  | 67 | 24.5(19.7-29.9) |  |
| Occupation | casual wage labourer | 9 | 3.4(1.7-6) | 0.2 | 83 | 31(25.7-36.7) | 0.5 |
|  | housewife | 6 | 3.6(1.5-7.3) |  | 41 | 24.6(18.5-31.5) |  |
|  | not applicable/Not Employed/Student/others | 3 | 12.5(3.6-29.7) |  | 6 | 27.3(12.3-47.8) |  |
|  | salaried employee | 2 | 8(1.7-23.3) |  | 10 | 40(22.7-59.4) |  |
|  | self employed | 6 | 7.1(3-14.1) |  | 23 | 27.4(18.7-37.6) |  |
| Wealth Index | QU1 | 3 | 2.5(0.7-6.6) | 0.54 | 22 | 18.5(12.3-26.2) | 0.001 |
|  | QU2 | 4 | 3.2(1.1-7.5) |  | 26 | 21.1(14.6-29) |  |
|  | QU3 | 7 | 5.1(2.3-9.8) |  | 44 | 32.6(25.1-40.8) |  |
|  | QU4 | 6 | 6.3(2.7-12.4) |  | 40 | 41.7(32.2-51.7) |  |
|  | QU5 | 6 | 6.5(2.7-12.8) |  | 31 | 33.3(24.4-43.3) |  |

The prevalence of diabetes increased with increasing age, male gender and more among non tribes as shown in the above table. There was statistically significant association of hypertension in the tribal area with increasing age, illiteracy and higher wealth.

**S4 Table. Association of diabetes and hypertension with tobacco and alcohol use (Univariate) among all study subjects**

|  | | DM | | | HTN | | |
| --- | --- | --- | --- | --- | --- | --- | --- |
|  | | n | % |  | n | % |  |
| Ever used smoked tobacco | No | 12 | 3.2(1.8-5.4) | 0.03 | 96 | 25.8(21.6-30.4) | 0.03 |
|  | Yes | 14 | 7.2(4.2-11.5) |  | 67 | 34.5(28.1-41.4) |  |
| smoking | Never smoked | 12 | 3.2(1.8-5.4) | 0.03 | 96 | 25.9(21.6-30.5) | 0.1 |
|  | Past smoker | 4 | 4.7(1.6-10.7) |  | 29 | 33.7(24.4-44.1) |  |
|  | Current smoker | 10 | 9.2(4.8-15.7) |  | 38 | 34.9(26.4-44.1) |  |
| Daily smoking | Never smoked | 12 | 3.2(1.8-5.4) | 0.07 | 96 | 25.9(21.6-30.5) | 0.2 |
|  | Smoked in past | 4 | 4.7(1.6-10.7) |  | 29 | 33.7(24.4-44.1) |  |
|  | Current occasional smoker | 1 | 12.5(1.4-45.4) |  | 3 | 37.5(11.9-70.5) |  |
|  | Current daily smoker | 9 | 8.9(4.5-15.6) |  | 35 | 34.7(25.9-44.3) |  |
| Passive smoking | No | 13 | 4(2.2-6.5) | 0.4 | 99 | 30.4(25.6-35.5) | 0.3 |
|  | Yes | 13 | 5.4(3.1-8.8) |  | 64 | 26.7(21.4-32.5) |  |
| daily use of smokeless tobacco | Never used | 23 | 4.8(3.1-6.9) | 0.7 | 137 | 28.4(24.5-32.6) | 0.7 |
|  | past user | 0 | 0(.-.) |  | 7 | 38.9(19.4-61.7) |  |
|  | Occasional current user | 0 | 0(.-.) |  | 1 | 16.7(1.9-55.8) |  |
|  | Daily current user | 3 | 5(1.4-12.7) |  | 18 | 30(19.5-42.3) |  |
| Ever used smokeless tobacco | No | 23 | 4.8(3.1-6.9) | 0.6 | 137 | 28.4(24.5-32.6) | 0.6 |
|  | Yes | 3 | 3.6(1-9.2) |  | 26 | 31(21.8-41.4) |  |
| Overall tobacco use | Never used tobacco | 12 | 3.2(1.8-5.4) | 0.1 | 96 | 25.9(21.6-30.5) | 0.2 |
|  | Past user | 2 | 6.7(1.4-19.7) |  | 12 | 40(24-57.8) |  |
|  | Current chewer of tobacco only | 2 | 3.6(0.8-11.2) |  | 17 | 30.9(19.9-43.9) |  |
|  | Current smoker only | 9 | 9.1(4.6-15.9) |  | 36 | 36.4(27.4-46.1) |  |
|  | Current smoker and chewer of tobacco | 1 | 10(1.1-38.1) |  | 2 | 20(4.4-50.3) |  |
| ever used alcohol | No | 20 | 4.2(2.7-6.3) | 0.4 | 126 | 26.8(22.9-30.9) | 0.017 |
|  | Yes | 6 | 6.3(2.7-12.6) |  | 37 | 38.9(29.6-49) |  |
| Current alcohol use | Never used alcohol | 20 | 4.2(2.7-6.3) | 0.7 | 126 | 26.8(22.9-30.9) | 0.054 |
|  | Current user | 4 | 6.1(2.1-13.8) |  | 25 | 37.9(26.9-49.9) |  |
|  | Past user of alcohol | 2 | 6.9(1.5-20.3) |  | 12 | 41.4(25-59.4) |  |
| Daily alcoholc use | Not current user | 22 | 4.4(2.8-6.4) | 0.14 | 138 | 27.5(23.8-31.6) | 0.15 |
|  | Not daily user | 3 | 4.9(1.4-12.5) |  | 24 | 39.3(27.8-51.9) |  |
|  | Daily user | 1 | 25(2.8-71.6) |  | 1 | 25(2.8-71.6) |  |
| HRD | Not current alcohol user | 23 | 4.6(3-6.7) | 0.9 | 139 | 27.7(24-31.8) | 0.2 |
|  | <6 SD | 2 | 5.4(1.1-16.2) |  | 12 | 32.4(19.1-48.4) |  |
|  | >6SD | 1 | 3.6(0.4-15.5) |  | 12 | 42.9(26-61.1) |  |

Diabetes was associated with smoking at least once in life, in that also, being current smoker has higher risk. There was statistically significant association of hypertension in the tribal area with ever use of tobacco and ever use of alcohol.

**S5 Table. Association of diabetes and hypertension with dietary pattern (Univariate) among all study subjects**

|  | | DM | | | HTN | | |
| --- | --- | --- | --- | --- | --- | --- | --- |
|  | | n | % | p | n | % | p |
| Inadequate fruits and vegetables | No | 1 | 6.7(0.7-27.2) | 0.7 | 1 | 6.7(0.7-27.2) | 0.06 |
|  | Yes | 25 | 4.5(3-6.5) |  | 162 | 29.4(25.7-33.3) |  |
| Added salt use frequency | Never | 17 | 3.9(2.4-6.1) | 0.1 | 121 | 28(23.9-32.4) | 0.6 |
|  | Rarely | 2 | 4(0.8-12.2) |  | 15 | 30.6(19.1-44.3) |  |
|  | Sometime | 2 | 5.7(1.2-17.1) |  | 14 | 40(25.1-56.5) |  |
|  | often | 5 | 13.2(5.2-26.5) |  | 9 | 23.7(12.4-38.8) |  |
|  | Always | 0 | 0(.-.) |  | 4 | 33.3(12.5-61.2) |  |
| Processed food use frequency | Never | 4 | 3.9(1.3-9.1) | 0.5 | 32 | 31.4(23-40.8) | 0.3 |
|  | Rarely | 2 | 2.4(0.5-7.3) |  | 19 | 22.6(14.7-32.4) |  |
|  | Sometime | 11 | 5.8(3.1-9.7) |  | 63 | 33.2(26.8-40.1) |  |
|  | often | 5 | 3.6(1.4-7.7) |  | 36 | 25.9(19.2-33.6) |  |
|  | Always | 4 | 7.8(2.7-17.6) |  | 13 | 25.5(15.1-38.6) |  |
| Consumption of butter/ghee | Never | 11 | 4.2(2.2-7.1) | 0.3 | 72 | 27.4(22.3-33) | 0.9 |
|  | Rarely | 8 | 8.6(4.2-15.6) |  | 28 | 30.1(21.5-39.9) |  |
|  | Sometime | 3 | 4.4(1.3-11.3) |  | 20 | 29.4(19.6-40.9) |  |
|  | often | 3 | 4.2(1.2-10.8) |  | 22 | 31(21.2-42.3) |  |
|  | Always | 1 | 1.4(0.2-6.4) |  | 21 | 29.6(19.9-40.8) |  |
| outside meals per week | Nil | 16 | 4.3(2.6-6.7) | 0.4 | 114 | 30.8(26.3-35.7) | 0.3 |
|  | 1-3 per week | 8 | 4.5(2.1-8.3) |  | 43 | 24.2(18.3-30.8) |  |
|  | >3 per week | 2 | 11.1(2.4-31.1) |  | 6 | 33.3(15.3-56.3) |  |
| Consumption of eggs | Never | 7 | 5(2.3-9.6) | 0.6 | 43 | 31.2(23.9-39.2) | 0.3 |
|  | Rarely | 2 | 9.5(2-27.2) |  | 9 | 42.9(23.7-63.8) |  |
|  | Sometime | 1 | 1.5(0.2-6.9) |  | 16 | 24.2(15.2-35.5) |  |
|  | often | 10 | 5.1(2.7-8.9) |  | 49 | 25.1(19.4-31.6) |  |
|  | Always | 6 | 4.1(1.7-8.3) |  | 46 | 31.5(24.4-39.3) |  |
| Consumption of fried food | Never | 1 | 2.7(0.3-11.9) | 0.4 | 12 | 32.4(19.1-48.4) | 0.6 |
|  | Rarely | 2 | 2.9(0.6-9) |  | 22 | 32.4(22.1-44) |  |
|  | Sometime | 11 | 7.1(3.9-12) |  | 49 | 31.8(24.9-39.5) |  |
|  | often | 11 | 4.5(2.4-7.6) |  | 62 | 25.4(20.3-31.1) |  |
|  | Always | 1 | 1.6(0.2-7.2) |  | 18 | 28.6(18.6-40.5) |  |
| Consumption of red meat | Never | 10 | 6.7(3.5-11.6) | 0.5 | 38 | 25.5(19-32.9) | 0.004 |
|  | Rarely | 3 | 6.5(1.9-16.4) |  | 11 | 24.4(13.7-38.3) |  |
|  | Sometime | 2 | 2.3(0.5-7.3) |  | 19 | 22.1(14.3-31.7) |  |
|  | often | 10 | 3.9(2-6.8) |  | 77 | 30.3(24.9-36.2) |  |
|  | Always | 1 | 3.1(0.3-13.7) |  | 18 | 56.3(39.1-72.3) |  |
| Consumption of fish | Never | 14 | 5.4(3.1-8.7) | 0.8 | 69 | 26.7(21.6-32.4) | 0.9 |
|  | Rarely | 8 | 4.9(2.4-9.1) |  | 49 | 30.4(23.7-37.8) |  |
|  | Sometime | 2 | 2.7(0.6-8.5) |  | 23 | 31.5(21.7-42.7) |  |
|  | often | 2 | 3.2(0.7-9.8) |  | 19 | 30.2(19.9-42.2) |  |
|  | Always | 0 | 0(.-.) |  | 3 | 27.3(8.3-56.5) |  |
| Consumption of chicken | Never | 8 | 5.5(2.6-10.1) | 0.6 | 45 | 30.8(23.8-38.6) | 0.03 |
|  | Rarely | 1 | 6.7(0.7-27.2) |  | 6 | 42.9(20.3-68.1) |  |
|  | Sometime | 1 | 1.3(0.1-6) |  | 13 | 17.1(9.9-26.7) |  |
|  | often | 15 | 5.1(3-8.1) |  | 83 | 28.3(23.4-33.7) |  |
|  | Always | 1 | 2.7(0.3-11.9) |  | 16 | 43.2(28.3-59.2) |  |
| Consumption of aerated drinks | Never | 14 | 7.3(4.3-11.7) | 0.2 | 57 | 30.2(24-37) | 0.5 |
|  | Rarely | 3 | 2.3(0.7-6.1) |  | 32 | 24.8(18-32.8) |  |
|  | Sometime | 4 | 3.6(1.2-8.3) |  | 34 | 30.6(22.6-39.6) |  |
|  | often | 5 | 4.2(1.6-9) |  | 32 | 27.1(19.7-35.6) |  |
|  | Always | 0 | 0(.-.) |  | 8 | 42.1(22.3-64.1) |  |
| Consumption of sweet drink | Never | 5 | 3.4(1.3-7.2) | 0.3 | 40 | 27.2(20.5-34.8) | 0.9 |
|  | Rarely | 8 | 6.3(3-11.5) |  | 38 | 29.9(22.5-38.3) |  |
|  | Sometime | 3 | 2.7(0.8-7) |  | 31 | 27.9(20.2-36.8) |  |
|  | often | 10 | 6.5(3.4-11.2) |  | 45 | 29.4(22.6-37) |  |
|  | Always | 0 | 0(.-.) |  | 9 | 32.1(17.2-50.5) |  |
| Physical activity level | Low | 2 | 11.8(2.5-32.7) | 0.3 | 7 | 43.8(22.2-67.4) | 0.2 |
|  | Moderate | 4 | 5.4(1.9-12.3) |  | 16 | 21.6(13.4-32) |  |
|  | High | 20 | 4.2(2.7-6.3) |  | 140 | 29.4(25.5-33.6) |  |

Diabetes is not associated with any of the dietary factors in this study. There was statistically significant association of hypertension in the tribal area with frequency of consumption of chicken and red meat.

**S6 Table. Association of diabetes and hypertension with anthropometry (Univariate) among all study subjects**

|  | | DM | | | HTN | | |
| --- | --- | --- | --- | --- | --- | --- | --- |
|  | | n | % | p | n | % | p |
| TBF | Normal | 12 | 2.8(1.6-4.7) | 0.001 | 97 | 23(19.2-27.2) | <0.001 |
|  | Increased(M- >26, F>32) | 13 | 9.6(5.5-15.5) |  | 63 | 46.7(38.4-55.1) |  |
| VF | <=9(normal) | 16 | 3.1(1.9-4.9) | <0.001 | 138 | 27(23.3-31) | 0.007 |
|  | 10-14(high) | 5 | 14.7(5.8-29.3) |  | 15 | 44.1(28.5-60.7) |  |
|  | >=15(Very high) | 4 | 33.3(12.5-61.2) |  | 7 | 58.3(31.2-82) |  |
| BMI category | Underweight(<18.5) | 4 | 1.6(0.6-3.8) | <0.001 | 36 | 14.8(10.7-19.6) | <0.001 |
|  | Normal (18.5-23) | 8 | 3.9(1.9-7.2) |  | 70 | 34.1(27.9-40.8) |  |
|  | Overweight (23.1-25) | 4 | 8.3(2.9-18.6) |  | 22 | 45.8(32.3-59.8) |  |
|  | Obese (>25) | 9 | 13.4(6.9-23.1) |  | 35 | 52.2(40.4-63.9) |  |
| WC | Normal | 9 | 2.2(1.1-4) | <0.001 | 94 | 23.2(19.2-27.4) | <0.001 |
|  | Increased | 17 | 10.9(6.7-16.5) |  | 68 | 43.6(36-51.4) |  |
| WHR category | Normal | 12 | 4.9(2.7-8.2) | 0.8 | 65 | 26.6(21.4-32.4) | 0.3 |
|  | Increased | 14 | 4.4(2.5-7.1) |  | 97 | 30.5(25.6-35.7) |  |

Diabetes is associated with higher total body fat, higher visceral fat, increased waist circumference and higher BMI. There was statistically significant association of hypertension in the tribal area with higher total body fat, higher visceral fat, , higher waist circumference, and higher BMI category.

**S7 Table. Association of diabetes and hypertension with demographic variables (Univariate) among tribal subjects**

|  |  | DM | | |  | HTN |  |
| --- | --- | --- | --- | --- | --- | --- | --- |
|  |  | n | Prevalence % | p | n | Prevalence % | p |
| Remoteness | Non remote | 4 | 3.3(1.1-7.7) | 0.4 | 36 | 30(22.4-38.6) | 0.1 |
|  | Somewhat remote | 3 | 3.2(0.9-8.3) |  | 18 | 19.6(12.5-28.5) |  |
|  | Remote | 1 | 0.8(0.1-3.8) |  | 39 | 32.2(24.4-40.9) |  |
| Age category in yrs | <25 | 3 | 3.9(1.1-10.2) | 0.053 | 7 | 9.5(4.3-17.7) | 0.001 |
|  | 26-40 | 3 | 1.7(0.5-4.6) |  | 46 | 26.6(20.4-33.5) |  |
|  | 41-55 | 0 | 0(.-.) |  | 29 | 43.9(32.4-56) |  |
|  | >=56 | 2 | 10(2.1-28.4) |  | 11 | 55(33.8-74.9) |  |
| Gender | Male | 5 | 4.7(1.8-10) | **0.06** | 37 | 34.9(26.3-44.3) | 0.052 |
|  | Female | 3 | 1.3(0.4-3.5) |  | 56 | 24.7(19.4-30.6) |  |
| Marital status | Married | 4 | 1.5(0.5-3.4) | 0.04 | 81 | 29.5(24.3-35) | 0.2 |
|  | Never married | 3 | 9.4(2.7-23) |  | 3 | 10(2.9-24.3) |  |
|  | seperated/Divorced | 0 | 0(.-.) |  | 1 | 33.3(3.9-82.3) |  |
|  | widowed | 1 | 4(0.4-17.2) |  | 8 | 32(16.4-51.5) |  |
| literacy | Illiterate | 3 | 1.7(0.5-4.4) | 0.4 | 54 | 30.2(23.8-37.2) | 0.3 |
|  | Literate | 5 | 3.2(1.2-6.9) |  | 39 | 25.3(19-32.6) |  |
| Occupation | casual wage labourer | 3 | 1.7(0.5-4.6) | 0.09 | 50 | 28.9(22.5-36) | 0.4 |
|  | housewife | 1 | 1.1(0.1-5) |  | 20 | 21.7(14.3-31) |  |
|  | not applicable/Not Employed/Student/others | 1 | 6.7(0.7-27.2) |  | 3 | 23.1(7-49.7) |  |
|  | salaried employee | 2 | 11.1(2.4-31.1) |  | 7 | 38.9(19.4-61.7) |  |
|  | self employed | 1 | 2.7(0.3-11.9) |  | 13 | 35.1(21.3-51.2) |  |
| Wealth Index | LOWEST* | 1 | 1.3(0.1-5.8) | 0.8 | 12 | 15.4(8.7-24.6) | 0.005 |
|  | SECOND | 3 | 3(0.9-7.8) |  | 23 | 23.2(15.8-32.2) |  |
|  | MIDDLE | 3 | 3.3(0.9-8.5) |  | 33 | 36.7(27.3-46.9) |  |
|  | FOURTH | 1 | 2.3(0.3-10.4) |  | 18 | 41.9(28-56.7) |  |
|  | HIGHEST | 0 | 0 |  | 7 | 30.4(14.8-50.7) |  |

*LOWEST- 1^st^ quintile of Wealth Index

None of the socio demographic variables were significantly associated with diabetes in tribal subjects. Gender is marginally significant, hence will be taken into regression. Increasing age and increasing wealth index were significantly associated with hypertension in tribal subjects

**S8 Table. Association of diabetes and hypertension with tobacco and alcohol use (Univariate) among tribal subjects**

|  | | DM | | | HTN | | |
| --- | --- | --- | --- | --- | --- | --- | --- |
|  | | n | Row N % | p | n | Row N % | p |
| Ever used smoked tobacco | No | 4 | 2(0.7-4.7) | 0.6 | 49 | 25(19.3-31.4) | 0.2 |
|  | Yes | 4 | 2.9(1-6.8) |  | 44 | 32.1(24.7-40.2) |  |
| smoking | Never smoked | 4 | 2(0.7-4.7) | 0.5 | 49 | 25(19.3-31.4) | 0.3 |
|  | Past smoker | 1 | 1.6(0.2-7.1) |  | 19 | 29.7(19.6-41.6) |  |
|  | Current smoker | 3 | 4.1(1.2-10.6) |  | 25 | 34.2(24.1-45.6) |  |
| Current use of smoked tobacco | No | 5 | 1.9(0.7-4.1) | 0.3 | 68 | 26.2(21.1-31.7) | 0.2 |
|  | Yes | 3 | 4.1(1.2-10.6) |  | 25 | 34.2(24.1-45.6) |  |
| Daily smoking | Never smoked | 4 | 2(0.7-4.7) | 0.6 | 49 | 25(19.3-31.4) | 0.5 |
|  | Smoked in past | 1 | 1.6(0.2-7.1) |  | 19 | 29.7(19.6-41.6) |  |
|  | Current occasional smoker | 0 | 0(.-.) |  | 2 | 28.6(6.5-64.8) |  |
|  | Current daily smoker | 3 | 4.5(1.3-11.6) |  | 23 | 34.8(24.2-46.8) |  |
| Ever used smokeless tobacco | No | 7 | 2.6(1.2-5) | 0.7 | 74 | 27.3(22.3-32.8) | 0.6 |
|  | Yes | 1 | 1.6(0.2-7.3) |  | 19 | 30.6(20.2-42.8) |  |
| daily use of smokless tobacco | Never used | 7 | 2.6(1.2-5) | 0.9 | 74 | 27.3(22.3-32.8) | 0.6 |
|  | past user | 0 | 0(.-.) |  | 6 | 42.9(20.3-68.1) |  |
|  | Occasional current user | 0 | 0(.-.) |  | 1 | 16.7(1.9-55.8) |  |
|  | Daily current user | 1 | 2.4(0.3-10.6) |  | 12 | 28.6(16.7-43.3) |  |
| Current tobacco use | No | 4 | 1.8(0.6-4.3) | 0.4 | 56 | 25.9(20.4-32.1) | 0.3 |
|  | Yes | 4 | 3.4(1.2-7.9) |  | 37 | 31.6(23.7-40.4) |  |
| Overall tobacco use | Never used tobacco | 4 | 2(0.7-4.7) | 0.8 | 49 | 25(19.3-31.4) | 0.6 |
|  | Past user | 0 | 0(.-.) |  | 7 | 35(17.2-56.8) |  |
|  | Current chewer of tobacco only | 1 | 2.3(0.2-10.1) |  | 12 | 27.3(15.9-41.6) |  |
|  | Current smoker only | 3 | 4.3(1.2-11.1) |  | 24 | 34.8(24.4-46.5) |  |
|  | Current smoker and chewer of tobacco | 0 | 0(.-.) |  | 1 | 25(2.8-71.6) |  |
| ever used alcohol | No | 5 | 1.8(0.7-3.9) | 0.15 | 68 | 24.8(20-30.2) | 0.006 |
|  | Yes | 3 | 5.1(1.5-13) |  | 25 | 42.4(30.4-55.1) |  |
| HRD | Not current alcohol user | 7 | 2.4(1.1-4.6) | 0.6. | 78 | 26.7(21.9-32) | 0.4 |
|  | <6 SD | 1 | 4.5(0.5-19.3) |  | 8 | 36.4(18.9-57.1) |  |
|  | >6SD | 0 | 0(.-.) |  | 7 | 36.8(18.2-59.1) |  |

***HRD-High Risk Drinking , >6SD per sitting**

Alcohol use and tobacco use were not significantly associated with diabetes in tribal subjects

Ever alcohol use p=0.1, hence will be taken into regression model. ever used alcohol was significantly associated with hypertension, however, ever smoked also taken into regression, as it was marginally significant.

**S9 Table. Association of diabetes and hypertension with dietary pattern (Univariate) among tribal subjects**

|  |  | DM | | |  | HTN |  |
| --- | --- | --- | --- | --- | --- | --- | --- |
|  |  | n | Prevalence % | p | n | Prevalence % | p |
| Inadequate fruits and vegetables | No | 1 | 14.3(1.6-50.1) | 0.2 | 0 | 0(.-.) | 0.1 |
|  | Yes | 7 | 2.1(1-4.1) |  | 93 | 28.5(23.8-33.6) |  |
| Added salt use frequency | Never | 6 | 2.4(1-4.8) | 0.2 | 66 | 26.3(21.1-32) | 0.02 |
|  | Rarely | 0 | 0(.-.) |  | 10 | 34.5(19.3-52.6) |  |
|  | Sometime | 0 | 0(.-.) |  | 11 | 47.8(28.7-67.5) |  |
|  | often | 2 | 9.1(1.9-26.1) |  | 2 | 9.1(1.9-26.1) |  |
|  | Always | 0 | 0(.-.) |  | 4 | 50(19.9-80.1) |  |
| Processed food use frequency | Never | 2 | 3.4(0.7-10.6) | 0.2 | 14 | 24.1(14.6-36.2) | 0.16 |
|  | Rarely | 0 | 0(.-.) |  | 10 | 23.3(12.6-37.3) |  |
|  | Sometime | 1 | 0.8(0.1-3.8) |  | 43 | 36.1(27.9-45) |  |
|  | often | 3 | 3.5(1-9) |  | 21 | 24.4(16.3-34.2) |  |
|  | Always | 2 | 7.4(1.6-21.7) |  | 5 | 18.5(7.4-35.9) |  |
| outside meals per week | Nil | 3 | 1.4(0.4-3.6) | 0.14 | 65 | 30.1(24.3-36.4) | 0.5 |
|  | 1-3 per week | 4 | 3.7(1.3-8.6) |  | 25 | 23.6(16.3-32.3) |  |
|  | >3 per week | 1 | 9.1(1-35.3) |  | 3 | 27.3(8.3-56.5) |  |
| Consumption of fried food | Never | 0 | 0(.-.) | 0.5 | 3 | 18.8(5.6-42.1) | 0.3 |
|  | Rarely | 1 | 2.4(0.3-10.6) |  | 11 | 26.8(15.2-41.6) |  |
|  | Sometime | 4 | 4.7(1.6-10.8) |  | 31 | 36.5(26.8-47) |  |
|  | often | 2 | 1.3(0.3-4.2) |  | 37 | 24.7(18.3-32) |  |
|  | Always | 1 | 2.4(0.3-10.8) |  | 11 | 26.8(15.2-41.6) |  |
| Consumption of red meat | Never | 3 | 7.9(2.3-19.6) | 0.003 | 6 | 15.8(6.9-29.7) | 0.007 |
|  | Rarely | 3 | 10(2.9-24.3) |  | 9 | 31(16.6-49) |  |
|  | Sometime | 1 | 1.6(0.2-7.1) |  | 15 | 23.4(14.4-34.8) |  |
|  | often | 1 | 0.6(0.1-2.6) |  | 50 | 27.8(21.6-34.6) |  |
|  | Always | 0 | 0(.-.) |  | 13 | 59.1(38.5-77.5) |  |
| Consumption of chicken | Never | 2 | 5.4(1.1-16.2) | 0.24 | 11 | 29.7(16.9-45.6) | 0.044 |
|  | Rarely | 1 | 9.1(1-35.3) |  | 5 | 50(22.4-77.6) |  |
|  | Sometime | 0 | 0(.-.) |  | 8 | 14.8(7.3-26) |  |
|  | often | 5 | 2.4(0.9-5.2) |  | 58 | 28.2(22.3-34.6) |  |
|  | Always | 0 | 0(.-.) |  | 11 | 42.3(25-61.3) |  |
| Consumption of aerated drinks | Never | 5 | 4(1.5-8.5) | 0.5 | 34 | 27.6(20.3-36) | 0.7 |
|  | Rarely | 0 | 0(.-.) |  | 20 | 26.7(17.7-37.4) |  |
|  | Sometime | 2 | 2.9(0.6-9) |  | 23 | 33.3(23.1-45) |  |
|  | often | 1 | 1.7(0.2-7.8) |  | 13 | 22.4(13.2-34.3) |  |
|  | Always | 0 | 0(.-.) |  | 3 | 37.5(11.9-70.5) |  |
| Consumption of sweet drink | Never | 2 | 2.2(0.5-6.9) | 0.03 | 23 | 25.8(17.6-35.6) | 0.9 |
|  | Rarely | 0 | 0(.-.) |  | 19 | 25.7(16.8-36.4) |  |
|  | Sometime | 0 | 0(.-.) |  | 20 | 31.3(20.9-43.2) |  |
|  | often | 6 | 6.7(2.9-13.4) |  | 25 | 28.4(19.8-38.4) |  |
|  | Always | 0 | 0(.-.) |  | 6 | 33.3(15.3-56.3) |  |
| Physical activity level | Low | 0 | 0(.-.) | 0.9 | 3 | 37.5(11.9-70.5) | 0.3 |
|  | Moderate | 1 | 2.3(0.3-10.4) |  | 8 | 18.6(9.2-32.1) |  |
|  | High | 7 | 2.5(1.1-4.8) |  | 82 | 29.1(24-34.6) |  |

Use of red meat and use of sweet drink were significant variables, and will be included in the regression model. Frequency of use of chicken, red meat and addition of salt were significantly associated with the hypertension among tribal subjects.

**S10 Table. Association of diabetes and hypertension with anthropometry (Univariate) among all tribal subjects**

|  |  | DM | | |  | HTN |  |
| --- | --- | --- | --- | --- | --- | --- | --- |
|  |  | n | Prevalence % | p | n | Prevalence % | p |
| Total Body Fat | Normal | 3 | 1.1(0.3-2.9) | 0.002 | 62 | 22.6(18-27.9) | <0.0001 |
|  | Increased(M- >26, F>32) | 4 | 7.7(2.7-17.3) |  | 28 | 53.8(40.4-66.9) |  |
| Visceral Fat | <=9(normal) | 6 | 1.9(0.8-3.9) | 0.2 | 83 | 26.8(22.1-31.9) | 0.1 |
|  | >10-14(high) | 1 | 6.3(0.7-25.7) |  | 7 | 43.8(22.2-67.4) |  |
| Waist Circumference | Normal | 5 | 1.8(0.7-4) | 0.2 | 63 | 23.2(18.5-28.5) | <0.0001 |
|  | Increased | 3 | 4.8(1.4-12.4) |  | 30 | 48.4(36.3-60.7) |  |
| WHR category | Normal | 5 | 3.3(1.3-7.1) | 0.3 | 39 | 25.7(19.2-33) | 0.4 |
|  | Increased | 3 | 1.7(0.5-4.4) |  | 54 | 29.8(23.5-36.8) |  |
| BMI category | Underweight(<18.5) | 3 | 1.8(0.5-4.7) | 0.1 | 24 | 14.5(9.8-20.5) | <0.0001 |
|  | Normal (18.5-23) | 3 | 2.4(0.7-6.2) |  | 45 | 36(28-44.7) |  |
|  | Overweight (23.1-25) | 0 | 0(.-.) |  | 14 | 60.9(40.6-78.6) |  |
|  | Obese (>25) | 2 | 10.5(2.3-29.7) |  | 10 | 52.6(31.2-73.4) |  |

Total body fat was significant variable for diabetes mellitus. Increasing total body fat, increasing waist circumference, BMI, decreasing skeletal mass, were significantly associated with the hypertension in tribal subjects
